# Soil disturbance influences ectomycorrhizal fungal communities: an in-situ experiment under Mediterranean holm oak (*Quercus ilex* L.)

**DOI:** 10.1007/s00572-026-01302-2

**Published:** 2026-08-13

**Authors:** Montan Gautier, Marc-André Selosse, Elisa Taschen, Jean-Michel Bellanger, Adrien Taudière, Franck Richard

**Affiliations:** 1 Centre d’Ecologie Fonctionnelle et Evolutive, Univ Montpellier, CNRS, EPHE, IRD, INSERM, Montpellier, France; 2 Pépinières Tenoux, Valdoule, Provence-Alpes-Côte d’Azur, France; 3 Institut Systématique Evolution Biodiversité, Muséum National d’Histoire Naturelle, CNRS, Sorbonne Université, Paris, France; 4 Department of Plant Taxonomy and Nature Conservation, University of Gdansk, Wita Stwosza 59, Gdansk, 80-308 Poland; 5 Institut Universitaire de France, Paris, France; 6 Eco&Sols, CIRAD, INRAE, IRD, Institut AgroMontpellier, Univ Montpellier, Montpellier, Occitanie, France; 7 IdEst, Saint-Bonnet-de-Salendrinque, France

**Keywords:** Ectomycorrhizal communities, Soil disturbance, Mediterranean forests, Ecological strategies, Mammal foraging, Fungal succession

## Abstract

The functioning of temperate forests is based on a symbiosis linking trees to a vast diversity of ectomycorrhizal (EM) fungi. Although disturbance is a major driver of biodiversity patterns in these ecosystems, its effects on the fine-scale distribution and the short-term dynamics of EM fungal communities are still largely unknown. We used an in situ experiment combined with the analysis of the ITS rDNA region of EM root tips to compare the composition of fungal communities before and one year after small-scale soil disturbance simulating animal bioturbation. Disturbance induced a transient increase in EM root tip abundance, a 56.3% decrease in species richness and a marked composition turnover. One year after disturbance, the proportion of Ascomycota had increased by 38.5% to represent 49.3% of the total number of mycorrhizae. This shift was associated with an increase in Pezizales (Tuberaceae, Helvellaceae, Pezizaceae and Pyronemataceae) and a decrease in Russulaceae, Cortinariaceae and Hydnaceae. The results show that small-scale soil disturbances promote highly localized fungal successions, from late-colonizing Basidiomycota lineages to early-colonizing Ascomycota. In temperate ecosystems, asynchronous small-scale disturbances may contribute to ectomycorrhizal community richness (α-diversity) and spatial turnover (β-diversity) by generating fine-scale spatial heterogeneity within forests.

**Supplementary Information:**

The online version contains supplementary material available at 10.1007/s00572-026-01302-2.

## Introduction

The ectomycorrhizal (EM) symbiosis is the dominant mutualistic interaction between fungi and plant roots in temperate, boreal, and Mediterranean ecosystems (Smith and Read 2010; Van der Heijden et al. 2015). This interaction is pivotal for nutrient uptake by both types of partners, plant hosts providing carbohydrates to EM fungi and receiving water and soil nutrients from their fungal symbionts (Smith and Read 2010). Belowground, EM trees recruit their partners among a highly diversified pool of > 20 000 fungal species within Asco- and Basidiomycota (Tedersoo et al. 2010). This diversity of EM fungi encompasses a wide spectrum of fungal life forms, including conspicuous pileate-stipitate fruitbody forms, sequestrate fungi (the so-called truffle-like fungi), crust-like resupinate and coral lifeforms (Sanchez Garcia et al. 2020).

In temperate forests, soils are inhabited by highly diversified EM fungal communities, with hundreds of species co-occurring at the local scale (e.g., Richard et al. 2005; Walker et al. 2005), some associated with the roots of single host individuals (Bahram et al. 2011). As forests mature, EM communities become richer in species (Smith et al. 2002), by accumulating specialized taxa (Taudiere et al. 2015), mainly Basidiomycota, that can complete their nutrient uptake by mobilizing nitrogen through the hydrolysis of carbon polymers which accumulate in the organic surface soil horizons with forest age (Courty et al. 2005; Kohler et al. 2015; Lindahl and Tunlid 2015). As a consequence, the diversity and the availability of organic matter present in forest soils strongly influence the distribution of EM fungal communities at the local scale (Tedersoo et al. 2008; Courty et al. 2016). Together with dispersal randomness, this chemical facet of the environmental filtering contributes highly patchy EM communities at metric scale (Genney et al. 2006; Courty et al. 2016; Clemmensen et al. 2024).

The ectomycorrhizal symbiosis governs the belowground functioning of the dominant forest systems in the Mediterranean. In this region, evergreen oaks (*Quercus ilex*, *Q. coccifera*, *Q. suber*, etc.) cover millions of monospecific, even-aged stands, shaped by long term anthropic agro-sylvo-pastoral practices (Quézel and Médail 2003). As in temperate forests, the distribution and the chemical diversity of soil organic matter drives patchy patterns of EM diversity (Richard et al. 2005; Courty et al. 2016). In these species-poor plant communities, drought-adapted lineages of Asco- and Basidiomycetes interact belowground in species rich EM assemblies (Richard et al. 2011), with sequestrated life-forms dominating the EM communities in the driest habitats (Authier et al. 2025). In the Mediterranean as in most temperate forests, these symbionts play a pivotal role at the early-stage of ecological successions, through the production of long-lived spores which preferentially colonize tree seedlings after disturbance (e.g., *Rhizopogon spp*, Horton & Bruns 1999, Bruns et al. 2009, Avis et al. 2017; *Tuber spp*, Taschen et al. 2022; *Geopora spp*, Authier et al. 2025).

Ecological disturbance is a major driver of spatial patterns and temporal dynamics of diversity in nature (Connell 1978; Viljur et al. 2022). Depending on their life-history traits, living organisms adapt differently to these events that suddenly and unpredictably disrupt environmental conditions and modify resource availability. These species-dependent responses to disturbance distinguish ruderal strategies from competitive ones, the fitness of the former being favored by disturbance, that of the latter being reduced by disturbance (Grime 1977; Westoby et al. 2002; Büchi and Vuilleumier 2016). Initially theorized for vascular plants by Grime (1977) and subsequently extended to various lineages of organisms (Grime and Pierce 2012), the so-called Grime’s framework of ecological ruderal and competitive strategies has been applied to EM fungi in the 1980s, mainly in a context of host forest establishment and succession (Dahlberg and Stenlid 1990; Bonello et al. 1998; Bruns et al. 2002; see also Douhan et al. 2011 for a review).

Based on fruitbody successions in post-agricultural contexts, Last et al. (1987) tried to accommodate EM diversity in Grime’s model by distinguishing “Early-“ (e.g.,* Hebeloma*, *Inocybe*) from “Late-“ (e.g., *Russula*, *Cortinarius*) and “Multi-stage” fungi (e.g., *Cenococcum geophilum*, *Laccaria laccata*), depending on their ability to produce fruitbodies and/or establish as ectomycorrhizas during forest chrono-sequences. Since the publication of this pioneer work, a vast body of literature has been accumulated concerning the temporal dynamics of EM fungal communities during ecosystem development. A consistent trend, showing contrasted patterns among the main lineages of Basidio- and Ascomycota, from early- (e.g., *Cenococcum*, *Thelephora* and *Tomentella*) to late-successional (e.g., *Boletus*, *Russula* and *Lactarius*) genera, emerged from the gathered body of case studies (see Dickie et al. 2013 for a review) and suggesting the existence of contrasted ecological strategies within EM diversity. Similarly, the response of EM diversity to disturbances has been widely documented response of EM communities to a wide range of disturbances (see Kalucka & Jagodzinski 2017 for a review), during primary (e.g., Nara 2006 for volcanic desert, Jumpponen et al. 2012 for glacier forefronts, Ashkannejhad and Horton 2006 for sand dune) and secondary successions (see Taudiere et al. 2017 for a review concerning wildfires; Jones et al. 2003 for a review concerning forest logging; van der Linde et al. 2018 and Jörgensen et al. 2024 for nitrogen deposition). From this corpus, and despite the accumulation of data showing the existence of post-disturbance dynamics in ectomycorrhizal communities (Douhan et al. 2011), Grime’s theory applied to EM fungal communities should be more thoroughly investigated and tested in a broader range of ecological contexts.

In Mediterranean and temperate forests, soils under trees are exposed to intense foraging by a wide variety of animals, including large mammals such wild boars (*Sus scrofa* L.), whose bioturbation strongly impacts ecosystem processes worldwide (Larson et al. 2005). These animals overturn and mix soil horizons in search of small vertebrates, invertebrates, plant roots and seeds, and hypogeous mushrooms (Schley and Roper 2003; Hohmann and Huckschlag 2005), influencing pedogenetic processes (Don et al. 2019) and shaping biotic interactions (Whitford and Kay 1999). Mammal foraging for resources, in addition to promoting spore dispersal and affecting germination through passage in digestive system (see Elliott et al. 2022 for a review), strongly affects both chemical and physical facets of EM fungi’s habitats, because it mixes soil horizons, traps organic matter in excavations and improves water and air infiltration in soils (Eldridge and James 2009). While extensively documented for arbuscular mycorrhizal (AM) fungi (e.g., Jasper et al. 1991; McGonigle & Miller 1996; Hart and Reader 2004; Schnoor et al. 2011), and despite its major impact on the distribution and availability of critical resources, the effects of soil disturbance on EM fungi remain poorly documented so far (Cho et al. 2017; Kranabetter et al. 2017). To the best of our knowledge, no attempt to document the fine-scale spatio‐temporal effects of small-scale soil disturbance on EM fungal communities has been made to date (see Eldridge et al. 2015 for an analysis for microbial communities; Miranda et al. 2019 for endophytic fungi).

**Fig. 1:**
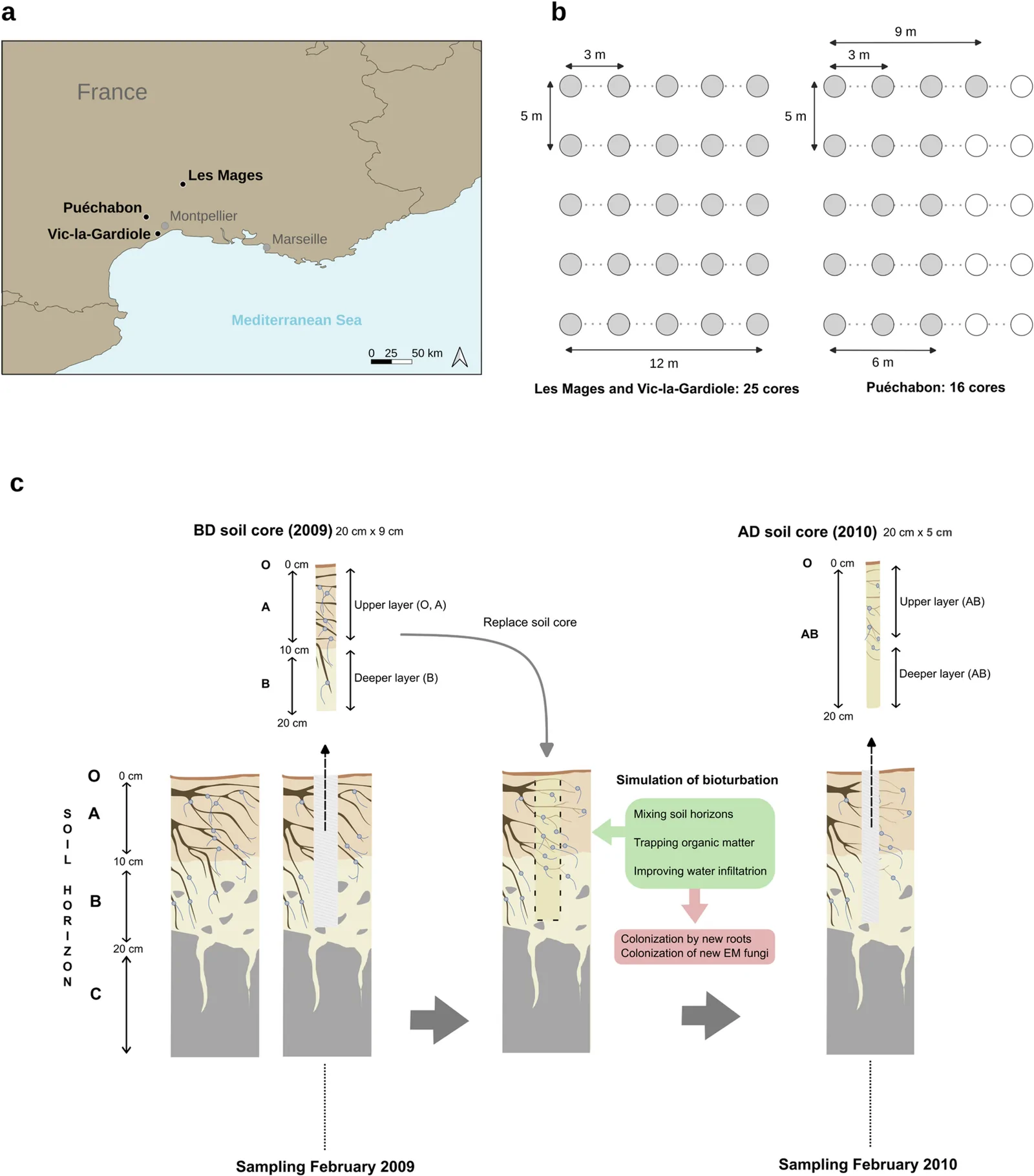
Representation of (**a**) the study sites, (**b**) the transect design and (**c**) the ingrowth core experiment used to study the response of ectomycorrhizal fungal communities to small-case disturbance. BD: before disturbance soil cores; AD: after disturbance soil cores. Blue dots symbolize ectomycorrhizal root tips

In this work, we used an in situ experiment to mimic natural bioturbations and evaluate their influence at a fine-scale on EM fungal communities in three *Quercus ilex* Mediterranean forests. According to Beca et al. (2022), the ingrowth core approach simulates bioturbations by mixing soil horizons, trapping organic matter and improving water infiltration (Fig. 1). We documented the response of EM fungi to soil disturbance by comparing the composition of fungal communities established on root tips of host trees just before and one year after soil coring. Our research hypothesis was that soil disturbance influences the composition and the richness of EM fungal communities, by favouring the establishment of ruderal species on newly produced root apices in ingrowth cores, at the expense of competitive species. We expected a turnover (i) in favour of groups of pioneer Ascomycota and few lineages of Basidiomycota (“early-stage fungi” *sensu* Last et al. 1987; Twieg et al. 2007; Teste et al. 2012), (ii) at the expense of lineages of highly specialized Basidiomycota (“late-stage fungi” *sensu* Last et al. 1987) with (iii) immediate negative impact on alpha diversity. Based on this experiment, we aimed to assess how natural bioturbation drives the EM fungal communities through the framework of Grime’s model, to improve our understanding of fungal ecological strategies.

## Materials and methods

### Sampling design

To evaluate the influence of the disturbance event on the temporal dynamics of EM community, complementary samplings were performed. First, the seasonal change in the composition of EM communities was documented at Puechabon site.

In this first experiment, four coring campaigns were carried out in December 2007, June 2008, December 2008 and June 2009. This diachronic survey was performed to provide a “without disturbance control” of the temporal dynamics of EM communities in the study system, and more specifically to assess the response of EM diversity to summer drought. At each of the four sampling dates, 45 soil cores were collected along three 4-m-long transects established in each of three sampling plots. The temporal patterns of EM communities were characterized by Sanger sequencing of EM root apices, as described in Richard et al. (2011), and using the same methodological approach as in the present study.

The present experiment specifically addressed the response of EM communities to small-scale disturbance. This experimental design did not include a synchronic undisturbed control, although the diachronic survey described above served as a proxy for an undisturbed control. The study was carried out at three sites in the French Mediterranean region, chosen across a 100 km long WSW-ENE oriented transect (Vic-la-Gardiole thereafter ‘Vic’ for easiness of read, Puéchabon, Les Mages; see Shahin et al. 2013 for details). At two of the three sites, Vic and Les Mages, 25 coring locations were selected within a large forest patch presenting similar composition (i.e., with *Q. ilex* as sole EM tree species), architecture (closed canopy successional stage, with no canopy gap) and structure (even-aged coppices; Shahin et al. 2013). At each site, soil cores were collected at 3 m intervals along five parallel 12 m-long transects distant by 5 m intervals to each other (Fig. 1). Because of strong field limitations at the Puechabon site due to the nature of its karstic soil system (see Rambal et al. 2003 for a detailed description), only 16 samples (from one 9 m-long and four 6 m-long transects) could be satisfactorily cored and analyzed.

The comparison between EM fungal communities established before and one year after soil disturbance was performed using an ingrowth core experiment (Hobbie 2006). This method was originally developed to estimate the annual root production under field conditions and the re-colonization of EM tips. By stimulating bioturbations by mixing soil horizons, trapping organic matter and improving water infiltration (Beca et al. 2022), this approach was used here as a proxy of natural bioturbations to explore the response of the EM communities to small-scale disturbance. Soils cores (20 cm deep × 9 cm diameter, hereafter called “Before Disturbance cores; BD”) were collected in February 2009 and transferred to the laboratory (Fig. 1), where they were divided into two layers: upper (0–10 cm deep) and deeper (10–20 cm deep) layers. At all sites, the upper layer was made of a superficial organic matter, and the deeper layer corresponded to a fissured bedrock with high volumetric rock content (see Shahin et al. 2013 for a description of soil characteristics). In each soil layer, all roots were carefully removed to characterize the EM fungal communities. Due to the very high density of EM root tips in some soil cores, EM abundance in these roots was estimated by counting a maximum of 20 EM root tips per soil layer and per core under a dissecting microscope to obtain a complete sampling of the diversity of morphotypes present in each soil horizon of each core. When fewer than 20 EM root tips were present, all EM root tips were collected. The two soil layers were pooled, and up to 20 EM root tips per core were then selected using a morphotype-based stratified strategy to maximize the diversity of morphotypes represented for molecular identification. All roots were removed from the soil, and all selected EM root tips were individually frozen, whereas the root-free soils of each core were returned to their original field location.

In February 2010, these soil samples were collected again to characterize their re-established EM fungal communities (Fig. 1), with a total of 1 125 EM root tips collected across both sampling dates for molecular identification. At this second sampling date, the core diameter was reduced to 5 cm (hereafter called “After Disturbance cores; AD”) and coring was targeted at the center of the primary 9 cm diameter sample. By taking only this subsample of soil, the method avoids any bias caused by the uncertain movement of the corer during sampling that may have collected soil from outside the disturbed zone (Makkonen and Helmisaari 1999; Lukac et al. 2003).

In order to quantitatively document the dynamics of EM colonization after disturbance, the number of picked EM root tips per layer per core was used as a proxy for abundance in BD and AD cores. In most BD cores, an exhaustive picking of EM root tips failed to reach 20. This pattern contrasted with AD cores, where 20 EM root tips were almost always systematically picked using this morphotype-based stratified strategy (Supplementary material 1).

To contextualize the post-disturbance response of EM communities with annual and seasonal natural dynamics at least at one site within the study system, we reanalyzed data from a previous study characterizing interannual seasonal changes at the same Puéchabon site (Richard et al. 2011). The composition of EM communities was characterized in December 2007 vs. December 2008 and in June 2008 vs. June 2009. At each of the four sampling dates, 45 soil cores were collected along three 4-m-long transects established in each of three sampling plots (Richard et al. 2011). The temporal patterns of EM communities were characterized by Sanger sequencing of EM root apices, as described in Richard et al. (2011), and using the same methodological approach as in the present study. This comparison provided an estimate of natural temporal variability in EM communities for one site within the study system.

### Molecular identification of ectomycorrhizal species

Fungal DNA was extracted from root tips with the REDExtract-N-Amp PCR ReadyMixTM kit (Sigma-Aldrich, St. Louis, US) with some modifications of the protocol as in Richard et al. (2011). The internal transcribed spacer (ITS) of the rDNA was amplified by PCR on 2 μL of freshly extracted DNA. PCR reactions were performed using the primer pair ITS1-F/ITS4 (Gardes & Bruns 1993) and amplicons were sent to AGOWA Genomics (Warwickshire, UK) for Sanger sequencing with the same primers. Fungal ITS sequences were edited with Sequencher v4.9 in 2010, grouped in OTUs at a 97% similarity threshold using the complete sequence dataset. A total of 114 representative sequences were deposited in Genbank under JF506742 to JF506855. 

A primary taxonomic assignment of these sequences was provided in the Supplementary Material of Shahin (2012), based on the annotation of closest sequences retrieved by a BLAST search at the NCBI (https://blast.ncbi.nlm.nih.gov/Blast.cgi#). We here revised these taxonomic assignments in three ways, on 2025 Dec 4th. First, we listed, for each published sequence, the best BLAST hit, defined as the GenBank sequence displaying the highest percentage of identity and a query cover above 70%. Second, we indicated the most inclusive and unambiguous Species Hypothesis (SHs) from UNITE v10.0 (https://unite.ut.ee/analysis.php, Kõljalg et al. 2005 ), together with its associated distance threshold (from 0.5% to 3%). Third, we manually inspected each of these SHs and merged their taxonomic content with that from step 1 (there is a delay in incorporating GenBank sequences in UNITE and in SHs), and with published or unpublished phylogenetic studies when available. The proposed name for each OTU indicated in Supplementary material 2 can thus be different from that of the best BLAST hit or from the most frequent annotation among the best BLAST hits, or from the name provided by UNITE for identified SHs (for a detailed methodology of annotation updates, see Supplementary material 3).

Overall, 103 published sequences were conserved in the present dataset, 80 of which are listed as reference sequences for distinct OTUs in Supplementary material 2. In addition to these published sequences, we completed our dataset with 23 sequences generated back in 2010 but not published by Shahin et al. (2013). The trophic guild status was assessed using FUNGuild (Nguyen et al. 2016), and only sequences assigned to EM fungi were retained. In total, the present work thus includes 103 OTUs with revised names at the species or the genus resolution. 

### Statistical analyses

To determine whether cores could be considered as spatially independent replicates in the analyses, tests for spatial autocorrelation were performed as follows: at each site, Bray–Curtis and Euclidean dissimilarity indices, respectively, were used to generate distance matrices among EM communities of different soil cores and geographical coordinates of the sampling locations. Mantel tests were performed using the ECODIST package (Goslee and Urban 2007) to test the effect of geographical distance between pairs of cores on EM community composition. The significance level was calculated by randomizing one matrix and calculating Mantel’s standardized r value for each random pairing as in Richard et al. (2005). Because no significant spatial autocorrelation (*P* > 0.1) was detected at each site and for each sampling date, cores were considered to be spatially independent replicates (see Shahin 2012 for details).

Differences in patterns of EM abundance among cores were tested using a generalized linear model (GLM) with a quasi-Poisson error distribution. Sampling date (BD vs. AD), site (Vic vs. Puéchabon vs. Les Mages) and soil depth (upper vs. deeper layer) were included as explanatory variables. To calculate Hill numbers, OTU abundance was estimated as the number of EM root tips assigned to each OTU at each sampling date and site. The Chao1 richness estimator was calculated using the vegan package (Oksanen et al. 2015). Differences among sites were tested using Tukey’s tests. To analyse general ecological trends of EM fungal communities, taxonomic assignments (phylum, order and family) were compiled for each taxon using taxize package with the NCBI Taxonomy database (Chamberlain and Szöcs 2013; Schoch et al. 2020) and abundances were pooled across soil depth. Variation in relative abundance of EM fungal families and genera between the two sampling dates was tested using Fisher’s tests, with P-values adjusted using the Bonferroni correction. Rarefaction curves were generated using the iNEXT package (Hsieh et al. 2016). All statistical tests were considered significant at *P* < 0.05.

Data analyses were performed using R Development software (V 4.4.2, R Core team 2024) and Rstudio environment (V 2025.09.0 + 387, Posit team 2025). Figures were produced in R Development software (V 4.4.2) and subsequently edited with Inkscape (Inkscape Project 2020).

## Results

### Influence of soil disturbance on EM abundance

**Fig. 2:**
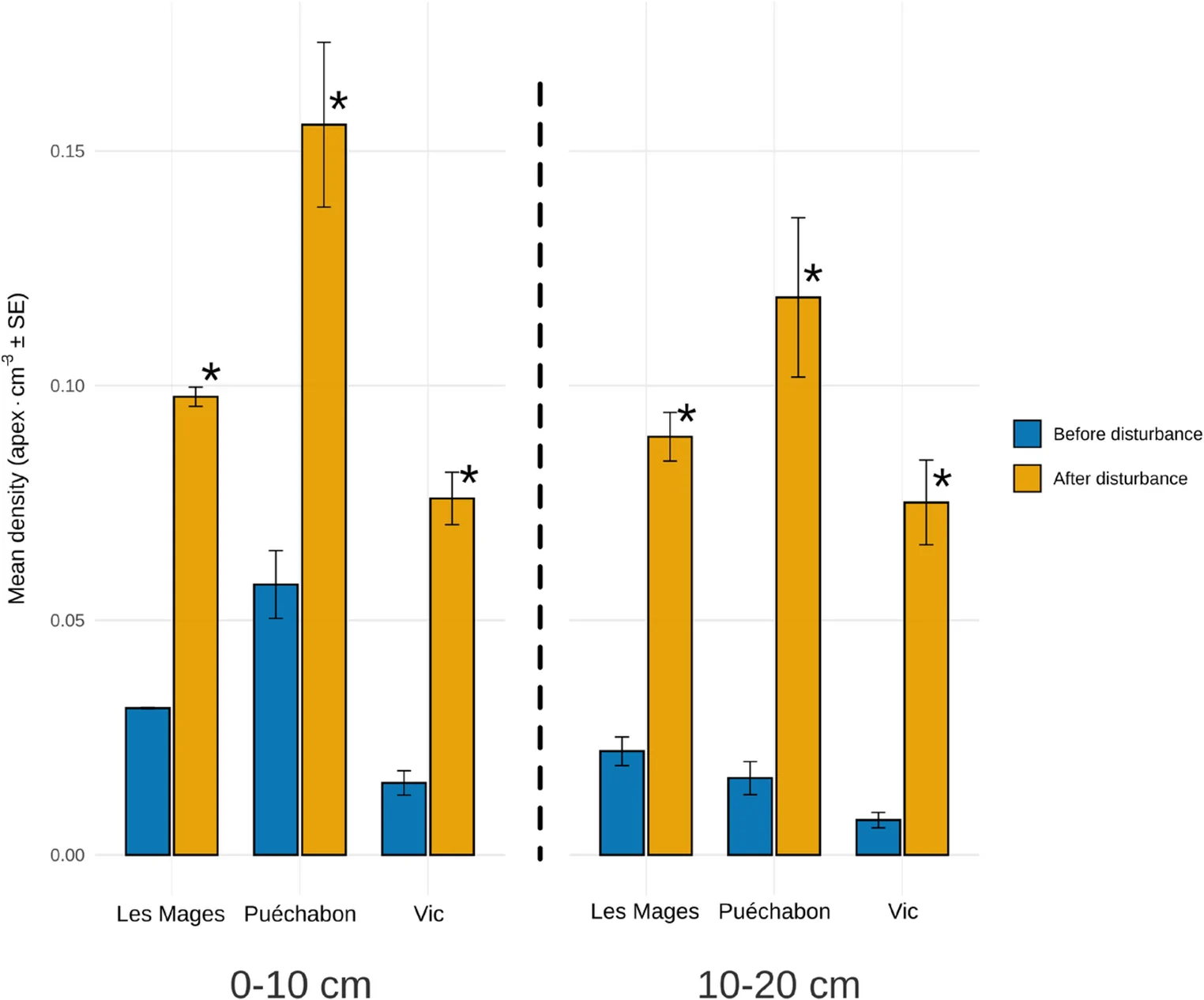
Mean density (± Standard Error) of ectomycorrhizal root tips (number of apeces. cm⁻³) at the three study sites in soils collected before (blue) and after (orange) disturbance and observed at two soil depths (0–10 cm and 10–20 cm). Asterisks indicate significant differences based on Tukey’s post hoc test (*p* < 0.001) within each site

Density of EM root tips significantly varied among sampling dates, sites and soil layers (Table 1). In both BD and AD, density was lowest at the Vic site (0.010 and 0.077 apex.cm⁻³, respectively) and highest at the Puéchabon site (0.049 and 0.150 apex.cm⁻³, respectively; Fig. 2). The Les Mages site displayed intermediate values, except in the deeper BD layer, which showed the highest value among sites (0.220 apex.cm-3; Fig. 2).

At all sites, the density of EM root tips was significantly higher in newly colonized (AD) than in initially collected (BD) cores, corresponding to a roughly 3.5-fold increase in mean EM root tip density (Table 1; Fig. 2). This increase rate was (i) significantly site-dependent (*P* < 0.001; analysis of deviance of the generalized linear model of the [site x sampling date] interaction) and (ii) significantly soil depth-dependent (*P* < 0.001; analysis of deviance of the generalized linear model of the [Soil depth x sampling date] interaction) (Table 1), a more pronounced coring impact in the deeper soil layer (+ 186.5% vs. + 533.3% in the upper and deeper soil layers, respectively; Fig. 2).

### Response of the EM community to soil disturbance

#### Influence of soil disturbance on alpha diversity

In all, 551 EM tips were successfully sequenced out of 1 125 and ascribed to 103 operational taxonomic units (OTUs). The remaining EM tips corresponded to sequencing failures or to sequences of such poor quality that they cannot be used for taxonomic analysis. Only one ectomycorrhiza matched a non-EM saprotroph (*Chaetasbolisia longiseta*) according to FUNGuild (Nguyen et al. 2016) and was excluded from the dataset. 87 OTUs (84.5% of the total number of OTUs) were found in BD, 38 (36.9%) were found in AD and 22 (21.4%) were found at both sampling dates.

**Fig. 3:**
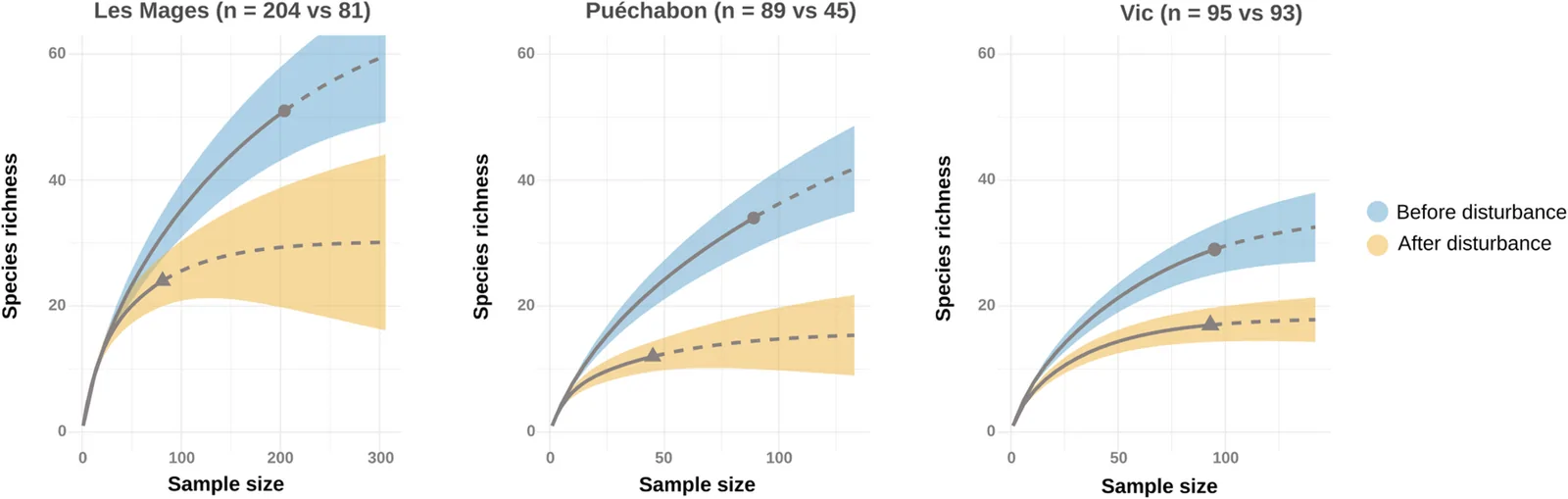
Species rarefaction curve of ectomycorrhizal richness at the three study sites, in soils collected before (blue) and after (orange) disturbance. Solid lines represent interpolated richness, and dashed lines represent extrapolated estimates beyond observed sample size. Shaded areas indicate 95% confidence intervals

Across all sites, EM fungal richness decreased after disturbance, with significant differences detected at 95% of confidence intervals (Fig. 3). When considering the normalized number of 75 EM sequences per site (Fig. 3; Table 2), the number of OTUs decreased from 29.9 to 23.4 (-21.7%), from 30.8 to 13.9 (-54.9%) and from 26.2 to 16.3 (-37.8%) at Les Mages, Puéchabon and Vic, respectively (Fig. 3; Table 2). Similar trends were observed for Chao1 richness, which decreased by 64.9% after disturbance across the whole dataset and consistently across individual sites.

Within the whole dataset, species richness (values of Hill number ^0^H) decreased by 56.3%, exponential of Shannon entropy (values of Hill number ^1^H) decreased by 43.9% and inverse Simpson diversity (values of Hill number ^2^H) decreased by 33.7% after disturbance (Table 2). This pattern was consistent with metrics of individual sites, except for Les Mages where inverse Simpson diversity increased by 13.3% (Table 2). Interestingly, Pielou’s evenness remained close to 1, with a 3.8% increase after disturbance when all sites were pooled. Similar trends were observed at individual sites, except at Vic-la-Gardiole, where evenness decreased by 6% (Table 2).

**Table 1:** Generalized linear model (quasi-Poisson) analysis of ectomycorrhizal (EM) root tips with site (Vic vs. Puéchabon vs. Les Mages), sampling date (before vs. after soil disturbance) and soil depth (0–10 cm vs. 10–20 cm) as variation factors. Significant differences (*P* < 0.05) among sites, between years, and between soil depths are shown in bold. Values in parentheses indicate the degrees of freedom for each factor and the residual degrees of freedom

	Site	Sampling Date	Soil depth	Site x Sampling Date	Site x Soil depth	Sampling Date x Soil depth	Residual deviance
	(2)	(1)	(1)	(2)	(2)	(1)	(159)
Total EM root tips							
*Deviance* *P*	0.7313 **< 0.001**	3.6724 **< 0.001**	0.0872 **< 0.001**	0.0914 **< 0.001**	0.0444 **< 0.05**	0.1069 **< 0.001**	1.0955

**Table 2:** Ectomycorrhizal fungal diversity expressed as Hill numbers (0H, 1H = , and 2H = 1/λ, where H’ is Shannon index and λ is the Simpson index) observed at the three study sites in soil collected before and after disturbance. The values in parentheses correspond to the estimated richness standardized to 75 EM sequences per site

Disturbance	Study site	Richness (^0^H)	Hill 1 (^1^H)	Hill 2 (^2^H)	Pielou’s evenness (J)	Chao1
BEFORE	Les Mages	51 (29.9)	23.9	12.8	0.81	69.1
	Puéchabon	34 (30.8)	20.4	11.8	0.86	55.4
	Vic	29 (26.2)	17.9	12.1	0.86	34
	All sites	87	42.8	25.5	0.84	117
AFTER	Les Mages	24 (23.4)	18.0	14.5	0.91	28.7
	Puéchabon	12 (13.9)	9.1	7.6	0.88	14
	Vic	17 (16.3)	10.1	6.5	0.81	17.6
	All sites	38	24.0	16.9	0.87	41.1

## Influence of soil disturbance on beta diversity

At the Puéchabon site documented in Richard et al. (2011), no significant differences were found between years (2007–2008 vs. 2008–2009) or between season (autumn vs. spring) at the phylum rank (Fisher’s tests, Bonferroni-adjusted *P* > 0.05; Supplementary material 4). Comparisons with BD revealed no significant differences (Fisher’s tests, Bonferroni-adjusted *P* > 0.05), except for autumn 2007 (Fisher’s tests, Bonferroni-adjusted *P* = 0.04). In contrast, all seasons differed significantly from AD (Fisher’s tests, Bonferroni-adjusted *P* < 0.01).

**Fig. 4:**
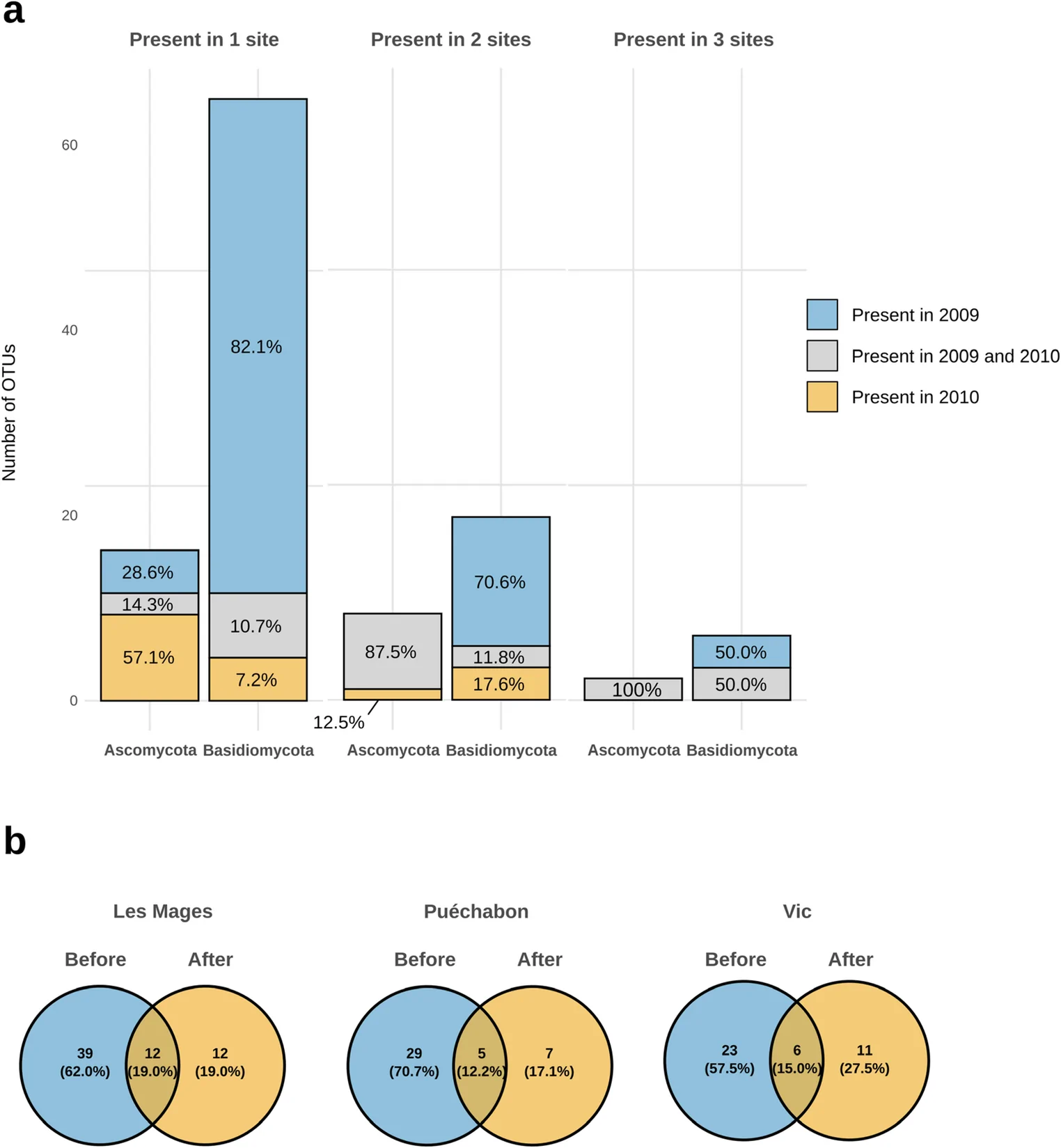
(**a**) Ectomycorrhizal fungal diversity and representativity across three study sites, showing the proportion of Asco- and Basidio- mycota OTUs detected in 2009, in 2010, or in both years. (**b**) Turnover of ectomycorrhizal OTUs from both fungal lineages (Ascomycota and Basidiomycota) at each study site before (blue) and after (orange) disturbance

In BD, three out of the 65 EM OTUs were detected at all three study sites, all belonging to Basidiomycota (Fig. 4a). The remaining OTUs were found at either one (50) or two (12) sites, including 92% and 100% of Basidiomycota, respectively (Fig. 4a). In AD, four out of the 16 EM OTUs were present at two sites, three of which belonged to Basidiomycota (Fig. 4a). The remaining OTUs were found at a single site (12), including 66.7% of Ascomycota (Fig. 4a). Across both dates, five out of the 22 EM OTUs were detected at all three study sites, including three Basidiomycota species (Fig. 4a). The remaining OTUs were found at either one (8) or two (9) sites, including 75% and 28.6% of Basidiomycota, respectively (Fig. 4a). At each study site, only twelve (19.0%), five (12.2%) and six (15.0%) OTUs were represented in both BD and AD at Les Mages, Puéchabon and Vic, respectively (Fig. 4b). In the same sites, the 87 OTUs found in BD accounted for 62.0% (39), 70.7% (29), and 57.5% (23) of the total number of OTUs, respectively (Fig. 4b). Conversely, the 38 OTUs detected in AD accounted for 19.0% (12), 17.1% (7) and 27.5% (11) of the total number of OTUs respectively (Fig. 4b).

Before disturbance, the EM community was dominated by Basidiomycota, with an Ascomycota/Basidiomycota (A/B) ratio of 0.13 on average (0.18 at Les Mages, 0.09 at Puéchabon, and 0.09 at Vic). Soil disturbance markedly reduced the relative Basidiomycota abundance and entailed a massive colonization by Ascomycota, with the A/B ratio increasing to 1.0 (increased by 5.9 times at Les Mages, 20 times at Puéchabon, and 7.7 times at Vic after disturbance).

**Fig. 5:**
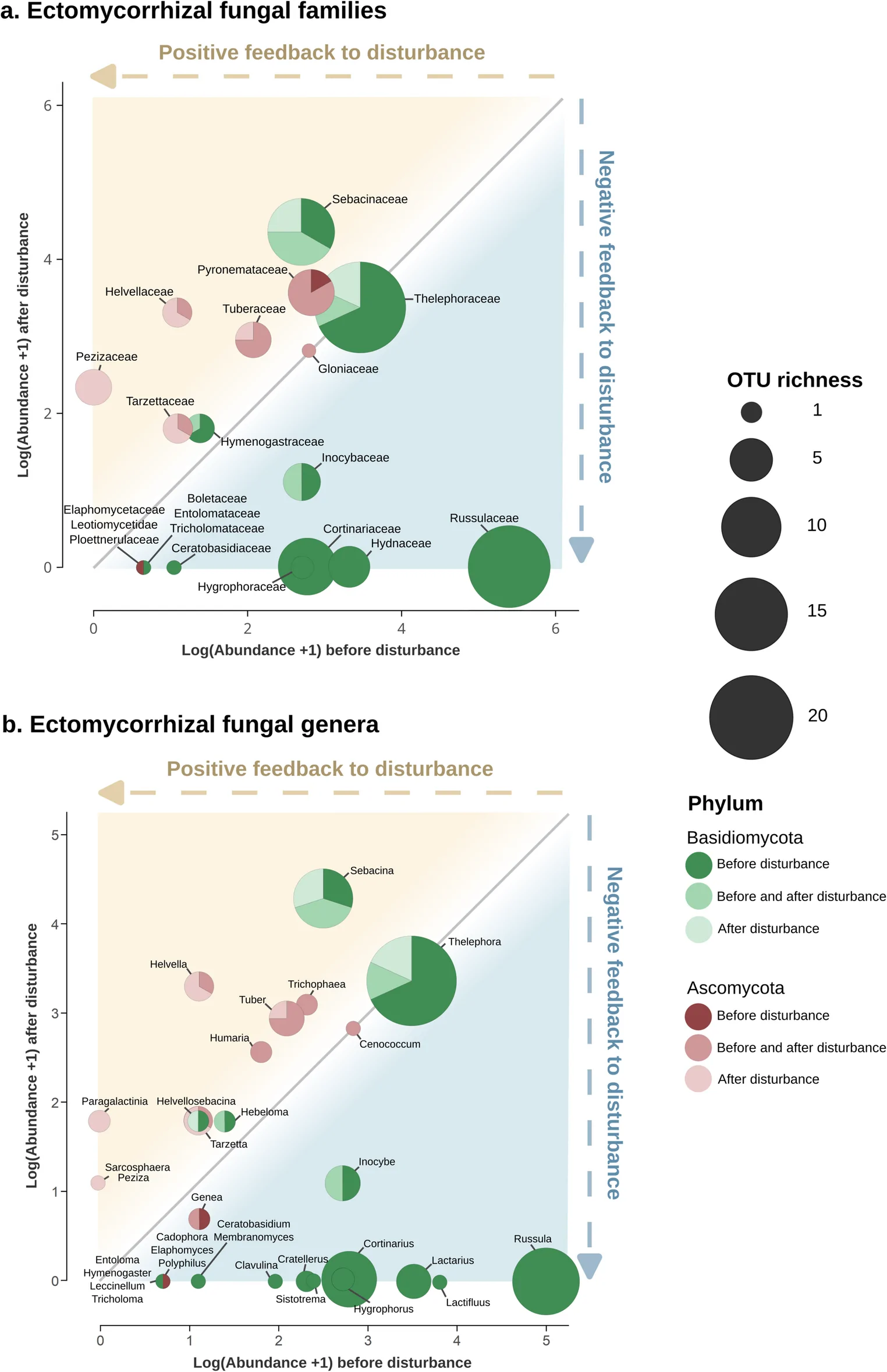
Position (in log + 1) of ectomycorrhizal fungal families (**a**) and genera (**b**) according to their abundance in soils collected before and after soil disturbance, with distinction between Asco- (pink) and Basidiomycota (green) lineages and indication of the corresponding number of OTUs

At the fungal family level, three patterns of response to soil disturbance were found (Fig. 5a). At all sites, Cortinariaceae, Hydnaceae and Russulaceae negatively responded to soil disturbance (Fisher’s tests, Bonferroni-adjusted *P* < 0.05; Fig. 5a, Supplementary material 5). These three families accounted for 67.3% of the EM root tips and 36.8% of the total number of OTUs in BD, with Russulaceae representing the most diverse (18 OTUs) and abundant (56.4% of EM root tips) one (Supplementary material 6). The latter family also displayed a strong shift in relative abundance after disturbance, dropping from 61.3%, 44.9%, 56.8% to 0% at Les Mages, Puéchabon and Vic sites, respectively. Eight other families (Boletaceae, Ceratobasidiaceae, Elaphomycetaceae, Entolomataceae, Hygrophoraceae, Leotiomycetidae, Ploettnerulaceae, and Tricholomataceae), poorly represented on root tips in BD, were not detected anymore in AD (Fisher’s tests, Bonferroni-adjusted *P* > 0.05; Fig. 5a; Supplementary material 5). Conversely, members of Sebacinaceae for Basidiomycota and Helvellaceae, Pezizaceae, Pyronemataceae and Tuberaceae for Ascomycota were significantly more abundant in AD than in BD (Fisher’s tests, Bonferroni-adjusted *P* < 0.05; Fig. 5a, Supplementary material 5, Supplementary material 6).

Sebacinaceae responded positively to soil disturbance at all sites, with relative abundances increasing from 4.4% to 28.4%, from 3.4% to 11.1%, and from 2.1% to 51.6% at Les Mages, Puéchabon and Vic sites, respectively. Similarly, Helvellaceae significantly increased its relative abundance from 1.0% to 14.8%, from 0% to 17.8%, and from 0% to 6.5% at the three sites, respectively.

The five remaining families (Gloniaceae and Tarzettaceae for Ascomycota, and Hymenogastraceae, Inocybaceae and Thelephoraceae for Basidiomycota) did not significantly vary in abundance between BD and AD (Fisher’s tests, Bonferroni-adjusted *P* > 0.05; Fig. 5a, Supplementary material 5, Supplementary material 6).

At a lower taxonomic rank, a marked decrease in abundance occurred after soil coring for three genera of Basidiomycota, including *Lactarius* (from 32 to 0 EM root tips), *Lactifluus* (from 43 to 0 EM root tips), *Russula* (from 144 to 0 EM root tips). Conversely, an increase was observed for three genera of Ascomycota, including *Helvella* (from 2 to 26 EM root tips), *Trichophaea* (from 9 to 21 EM root tips) and *Tuber* (from 7 to 18 EM root tips), as well as for one genera of Basidiomycota, *Sebacina* (from 11 to 71 EM tips). These differences were statistically significant (Fisher’s tests, Bonferroni-adjusted *P* < 0.05; Fig. 5b, Supplementary material 7). The *Cenococcum*, *Genea*,* Hebeloma*,* Helvellosebacina*, *Humaria*, *Inocybe*, *Tarzetta* and *Thelephora* genera did not significantly vary after disturbance. The three genera *Peziza*,* Sarcosphaera* and *Paragalactinia* were only detected in AD and conversely, the 14 genera *Cadophora*, *Ceratobasidium*, *Clavulina*, *Cortinarius*, *Craterellus*, *Elaphomyces*, *Entoloma*, *Hygrophorus*, *Hymenogaster*, *Leccinellum*, *Membranomyces*,* Polyphilus*,* Sistotrema* and *Tricholoma* were only detected in BD.

At the species rank, 14 OTUs accounted for more than 2% of EM root tips in BD, including 11 Basidiomycota (eight OTUs in the Russulaceae, *Hygrophorus russula*,* Sebacina* sp. 9 and *Sebacina* cf. *incrustans*) and three Ascomycota (*Cenococcum geophilum*, *Helvella neopallescens*, *Trichophaea* sp. 2). Among these OTUs, eight OTUs of Russulaceae and *Hygrophorus russula* were absent in AD.

One year after soil disturbance, 17 OTUs accounted for more than 2% of EM root tips, including nine species of Basidiomycota (six OTUs in the Sebacinaceae, two OTUs in the Thelephoraceae and *Hebeloma sinapizans*) and eight species of Ascomycota (*Cenococcum geophilum*,* Helvella neopallescens*,* Humaria* sp. 1, *Humaria* sp. 2, *Trichophaea* sp. 2, *Trichophaea sp. 1*,* Tuber dryophilum* and *Tuber ferrugineum*).

## Discussion

Our study revealed the fast and marked response of EM communities to small-scale soil disturbance, which contrasted with the temporal patterns of EM communities at the Puéchabon site, considered at the phylum rank (Supplementary material 4; Richard et al. 2011). Indeed, in the “without disturbance” experiment, the proportion of Ascomycota and Basidiomycota remained stable among sampling dates, and close to the values observed in BD cores in the present study, ranging from 81.3 to 88.5% of Basidiomycota. This pattern contrasts with the massive colonization of roots by Ascomycota in soils collected after disturbance. A consistent pattern was strikingly observed across study sites, with early-colonizing ruderal Ascomycota and Sebacinaceae succeeding competitive lineages of Basidiomycota on *Quercus ilex* roots and massively establishing in disturbed soils.

### Soil disturbance drives the dynamics of EM symbiosis

Our data show that one year after small-scale disturbance, the abundance of EM root tips per soil volume significantly increased (Table 1; Fig. 2) whereas specific richness decreased (Table 1; Fig. 3). However, a limitation of our study is that AD samples were smaller in diameter than BD samples, which may have, to a limited extent, contributed to the lower species richness in AD than BD samples. Although richness patterns remained consistent after standardized sampling and rarefaction analyses, future experiments using ingrowth cores may be methodologically optimized by using identical core diameters (or identical sampled soil volumes) before and after disturbance, to limit sampling effects on species richness estimates. However, this pattern suggests a strong colonization by some EM fungi that were favored by disturbance. Moreover, because this metric is not standardized (e.g., by mycorrhizal colonization), this increase cannot separate whether the disturbance stimulated root production, ectomycorrhizal formation, or both. Nevertheless, two mechanisms may have acted synergistically to induce this general positive effect of soil disturbance on symbiotic root tips.

First, the production of fine roots may have been stimulated in disturbed soils, which porosity was increased by bioturbation. Such cascading effects have been documented on *Quercus ilex* seedlings grown in greenhouses, in relation with the ability of plants to positively respond to a decrease in resistance of soil to root penetration (Cubera et al. 2009). In situ observations of root growth dynamics showed that the physico-chemical changes induced by soil disturbance stimulated root production, and that one year was required to re-establish natural patterns of fine root dynamics (Nakahata and Osawa 2017). In the studied system, the clayey texture of soil and its high stone content (Shahin et al. 2013) may have promoted the amplitude of this positive effect of disturbance on EM root tip abundance.

Second, the production of EM fungal hyphae and rhizomorphs, consecutively to spore germination and/or vegetative colonization from yet established neighboring individuals, may have been favored by increased soil porosity (e.g., Skinner and Bowen 1974). Such a positive effect of soil disturbance is far from being common to all EM fungal lineages, but it could reasonably be expected for species of Tuberaceae recorded in the EM community (Taschen et al. 2022). Indeed, there is a consistent body of literature that documents soil tillage as an efficient practice to stimulate vegetative development of true truffles cultivated in orchards (e.g., Salerni et al. 2014 for *Tuber magnatum* and Taschen et al. 2022 for *T. melanosporum*). In *T. melanosporum*, the whole biological cycle, including spore germination, hyphal development and fruiting may be stimulated by small-scale disturbances (Murat et al. 2016; Taschen et al. 2022). For other lineages that positively responded to small-scale disturbance (e.g., Helvellaceae, Sebacinaceae), specific measurements based on qPCR may usefully document the temporal dynamics of soil mycelia in disturbed habitats, to better understand the mechanisms involved in their fast establishment on newly produced root tips (Gryndler et al. 2013; Castaño et al. 2017).

### Soil disturbance induces unfavorable conditions for the dominant genera of Basidiomycota

One of the most remarkable observations in this study is the sharp decline in EM diversity one year after disturbance (Fig. 3; Table 2), due to a drastic reduction of the number of Basidiomycota OTUs in disturbed soils, with a spectacular drop of Inocybaceae and an absence of Boletaceae, Ceratobasidiaceae, Cortinariaceae, Entolomataceae, Hydnaceae, Hygrophoraceae, Russulaceae and Tricholomataceae on newly produced fine roots (Fig. 5a, Supplementary material 5). In these lineages and within the one-year timeframe of our survey, 40 species, including 18 Russulaceae, disappeared on EM root tips after disturbance. The amplitude of the observed species turnover may be considered under the perspective of the age of the studied forest system. In centuries-old Mediterranean *Q. ilex* coppices, highly specialized EM taxa with extended catabolic abilities toward complex sources of nutrients accumulated across the soil profile (Lindahl and Tunlid 2015), represent a major part of the diversity (Courty et al. 2016). In such habitats, small-scale disturbances may primarily affect this part of the functional EM diversity, through its drastic effect on soil physico-chemical properties. The first family impacted by this marked turnover, Russulaceae, is a key EM lineage in temperate forests (Looney et al. 2018). This family provides many EM taxa in ecosystems dominated by oaks (e.g., Morris et al. 2008; Hughes et al. 2025), notably in the Mediterranean region (Richard et al. 2005). In the studied system, Russulaceae are consistently more represented in deep mineral soil layers from undisturbed soils (Shahin et al. 2013). This vertical soil partitioning is well illustrated by the distribution of dominant *Russula* species (e.g., *R. olivacea*, *R. quercilicis* & *R. foetens*) and *Lactarius* (e.g., *L. acerrimus*), that were only recorded in the nutrient-poor, little disturbed mineral soil horizon. Our results are consistent with previous works that documented highly structured EM communities in temperate (Genney et al. 2006) and boreal forest soils (Rosling et al. 2003), evidencing that the environmental filtering, in particular the organic-mineral gradient from surface to bedrock, determines the vertical spatial partitioning of EM fungi (Rosling et al. 2003; Genney et al. 2006).

**Fig. 6:**
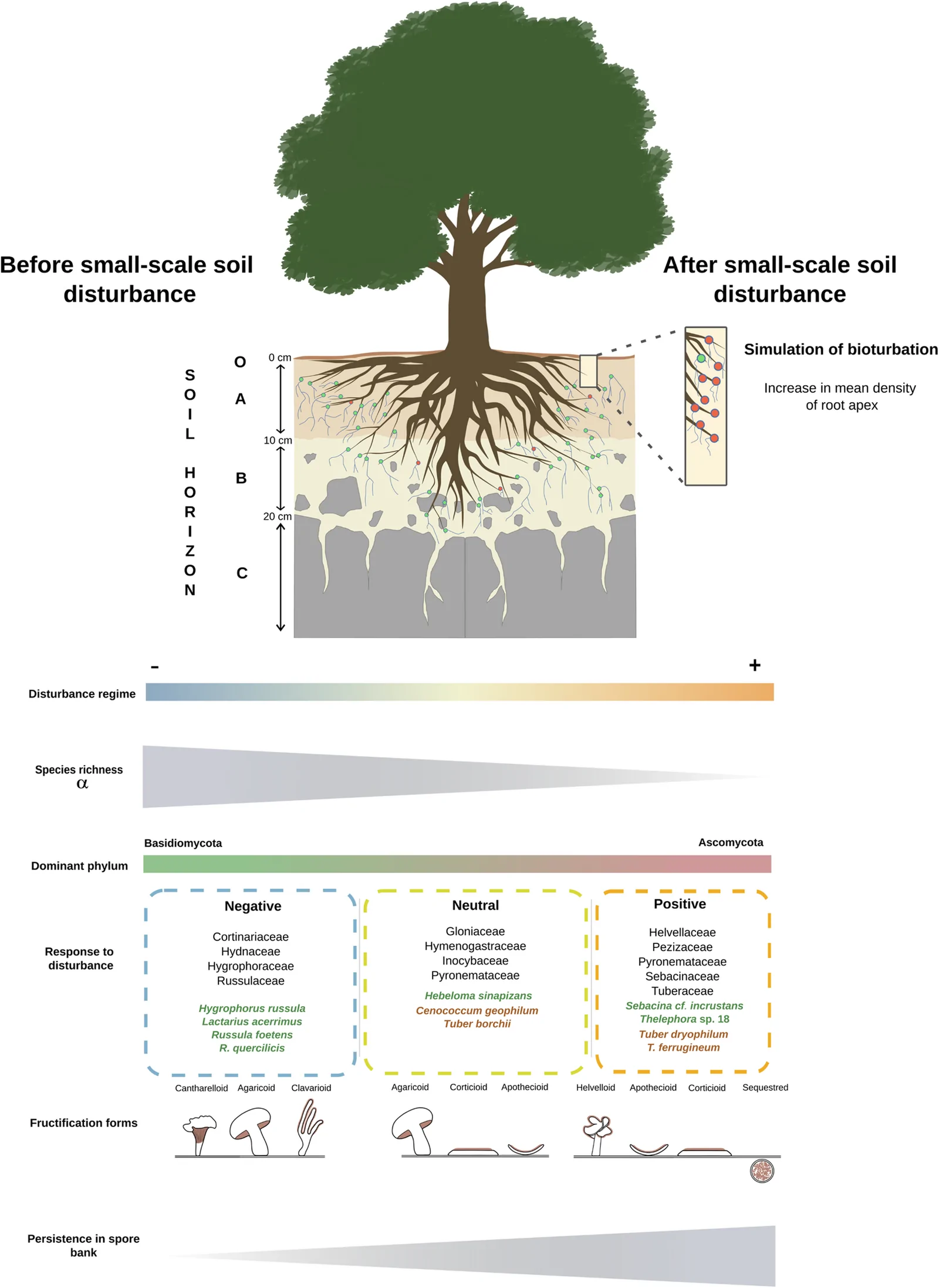
Scheme representing the functional diversity of EM fungi at various taxonomic levels in response to soil disturbance. Lineages and species of Asco- and Basidiomycota lineages are indicated in pink and green, respectively

This study suggests a mechanism which may drive the vertical distribution of EM fungal diversity in forest soils. Recurring disturbance of the organic surface horizon by forest fauna may selectively affect some major Basidiomycota lineages, including the species-rich Russulaceae (Fig. 6).

The sensitivity of Russulaceae to disturbance-mediated soil homogenization may explain its competitive ability with little ruderality *sensu* Grime’s (1977; late-stage *sensu* Last et al. 1987). The observed pattern may reflect acquired skills of the corresponding species to grow their mycelia in undisturbed patches (Visser 1995).

The mechanisms underlying the maintenance of competitive EM taxa in periodically bio-disturbed oak forest soils remain unclear. The highly erratic nature of disturbance regime may contribute to maintain undisturbed soil patches in these ecosystems. Indeed, in Mediterranean oak forests, acorn production shows highly variable patterns in space (Alejano et al. 2008; Koenig et al. 2013) and time (Garcia-Mozo et al. 2012). These heterogeneous patterns of resources availability may contribute to the persistence of undisturbed soil patches. This mechanism may favor the persistence of heterogeneous EM communities, and contribute to the extreme, highly documented and poorly understood EM species patchiness in these ecosystems (Courty et al. 2016). As a partial conclusion, understanding the close interaction between EM trees, their seed consumers and their associated EM symbionts, arises as a pivotal question to refine our understanding of the overall functioning of EM communities.

### Soil disturbance favors the establishment of Ascomycota and Sebacinaceae

The second notable result is the massive colonization of disturbed soils by Ascomycota at all sites. Rather than promoting the dominance of one or a few species, disturbance induced a structured turnover of EM communities, with several early-colonizing species replacing late-colonizing Basidiomycota while maintaining a relatively balanced distribution of abundances (Table 2). This pattern was mainly driven by the strong and positive response to disturbance of Pezizales species in the genera *Helvella*, *Humaria*, *Paragalactinia*, *Peziza*,* Trichophaea* and *Tuber* (Fig. 5b). The ruderal strategy unveiled here in Sebacinaceae is rather new. Members of this family are genuine EM fungi (Weiß et al. 2016), others associate with achlorophyllous orchids (Selosse et al. 2002) from old growth forests, but also dominant EM lineage in alpine areas above the tree line (Schön et al. 2022) so the family may have evolved different strategies depending on the species. Conversely, the affinity of many EM Pezizales for disturbed habitats has been widely documented by both fruiting-body emergence (e.g., Petersen 1985; Pfister and Healy 2021) and soil mycelia patterns (e.g. Pérez-Izquierdo et al. 2020). This positive response to disturbance has been firstly discussed in light of spore dispersal efficiency of the corresponding species (Caesar-TonThat et al. 2010; Manici et al. 2024).

This pattern is notably expected for some species belonging to Tuberaceae, whose cultivation in planted orchards includes soil tillage and spore dispersal as consensual practices to promote development of soil mycelia (Chevalier and Sourzat 2013; Salerni et al. 2014; Taschen et al. 2022). In the studied forests, *T. melanosporum* was not detected on *Q. ilex* fine roots, suggesting that closed canopy stable conditions were not favorable to this species (Bonito et al. 2013; Taschen et al. 2014). On the other hand, two species of true truffles, *T. rufum* and *T. dryophilum*, successfully established in disturbed soils, while a third one, *T. borchii*, was equally abundant in soils collected before and after disturbance (Supplementary material 2). Interestingly, *T. rufum* and *T. dryophilum* have been consistently reported as spontaneously co-occurring with *T. melanosporum* in planted truffle grounds, where they may benefit from plowing (Baciarelli-Falini et al. 2006).

This result sheds new light on the role of small-scale disturbance as a mechanism promoting species co-occurrence in pioneer EM fungal communities dominated by Ascomycota (Fig. 6). Spore banks are often enriched in sequestrate fungi such as truffles, which may be deposited at high concentrations in the soil profile (Glassman et al. 2015; Miller et al. 1994). Many of these spores are dispersed through animal mycophagy, and able to survive digestive tracts (Frank et al. 2009; Bonito et al. 2012). Consequently, spores persisting in the soil spore bank can rapidly germinate and colonize roots after disturbance, following ruderal and stress-tolerant strategies (Glassman et al. 2015). This colonization advantage stems from their higher germination efficiency and EM formation on host roots compared with more competitive fungi (Ishida et al. 2008; Nara 2009). Supporting this idea, a recent study using artificially disturbed microhabitats inoculated with pieces of *T. melanosporum* fruitbodies demonstrated that *T. melanosporum* ascospores can germinate and contribute to the sexual life cycle for 2–3 years after soil disturbance (Taschen et al. 2022). From the perspective of Grime’s model of ecological strategies, the disturbance responsiveness of Pezizales spores may thus be considered as a pivotal trait of pioneer EM fungal species (Fig. 6). Further studies specifically exploring the relationship between spore traits and ecological strategies in EM fungal communities may enlighten our knowledge of the evolutionary dimension of EM fungal successions.

### Thelephoraceae remain co-dominant after soil disturbance

The third marked result of the present research is the remarkable stability of Thelephoraceae upon soil disturbance, this family being dominant on *Q. ilex* fine roots before and after soil coring (Fig. 5a). This pattern arises from contrasted ecologies among species within the highly diversified genus *Thelephora* (incl. *Tomentella*), along a gradient of response to disturbance ranging from highly negative (e.g., *Thelephora* sp. 17) to highly positive (e.g., *Thelephora* sp. 18), including neutral responses from other taxa (Supplementary material 2). Evidence for the coexistence of such contrasting strategies within Thelephoraceae also emerges from published studies (see Taylor and Bruns 1999 for *T. sublilacina* a competitive species in mature forests; see Walker and Jones 2013 for *T. terrestris* a ruderal species in disturbed habitats). This pattern may also reflect ongoing taxonomic instability and partial phylogenetic overlap within Thelephoraceae (Kõljalg et al. 2005).

Interestingly, previous work conducted in the same ecosystem at one of our study sites (Puechabon) highlighted the functional diversity within Thelephoraceae when considering the response of the family to an increased drought (Richard et al. 2011). After 5 years of rainfall exclusion, Thelephoraceae remained the dominant family on *Q. ilex* fine roots (Richard et al. 2011), with contrasted response to drought depending on species, including positive (e.g., *T. lilacinogrisea*), negative (e.g., *T. subtestatea*) and neutral (e.g., *T. bryophila*). Altogether, the high taxonomic and functional diversity of Thelephoraceae constitutes an ideal model for further research on the evolutionary plasticity of ecological strategies in EM fungi, and may be one driving factor of their dominance in EM communities.

### Small-scale disturbance, an underestimated driver of EM fungal diversity?

So far, no study has directly examined the effect of small-scale soil disturbance, as a proxy of bioturbation by mammals on EM fungal communities. Of course, the ingrowth core experiment was used as a proxy that differs from foraging activity by wildlife in several respects (e.g., standardized depth and surface area, systematic root breakage, and backfilling with root-free soil). The results presented here should therefore be interpreted with the understanding that the patterns observed may have been influenced by these limitations of the method used. Natural bioturbators modify habitat characteristics by breaking the soil layers, increasing water infiltration, soil moisture, and the capture of organic matter (Miranda et al. 2019; Beca et al. 2022; Fig. 1). These animals act as ecosystem engineers by contributing to the patchiness of bacterial and fungal communities (Hawkins et al. 1996; Eldridge et al. 2015), with more hypogeous EM fungi inside enclosure of diggings mammals (Dundas et al. 2018). However, such patterns can result from different processes, such as spore dispersion by mycophagy or ectozoochory by bioturbators (Claridge and May 1994; Vašutová et al. 2019).

Our research highlights the underestimated role of soil disturbance in maintaining the hyper-diversity and the hyper-patchiness of EM fungal communities at the local scale in Mediterranean ecosystems (Fig. 6), by favoring the maintenance of (1) poorly represented lineages of ruderal Ascomycetes (e.g.,*Tuber*, *Helvella*) and (2) highly adapted Basidiomycota in species-rich genera (e.g., *Thelephora*, *Sebacina*). Indeed, bioturbation allows ruderal strategies to coexist with competitive strategies in dense canopy cover and mature forests, contributing to the high diversity of EM fungal communities.

## Conclusions

By integrating Grime’s framework of ecological strategies into the dynamics of EM communities, this study contributes to extending to EM fungi the biological and taxonomical bases of this conceptual theory, originally elaborated for vascular plants. Here we show why soils under mature forests can host a fine-scale mosaic of EM fungal communities which undergo rapid species turnover in response to bioturbation.

Under closed canopies of mature stands, a kind of post-disturbance belowground EM successions occur independently of the aging of the forest and associated parameters. Our study sheds new light on the ecology of the iconic true truffles, whose life-history traits not only include their aptitude to manipulate the mammals which disperse their spores, but also their ability to benefit from the disturbances they indirectly cause through the manipulated fauna, at the expense of the EM species with which they compete.

## Supplementary Information

Below is the link to the electronic supplementary material.

Supplementary Material 1

## Data Availability

All sequences are deposited in GenBank (NCBI), with accession numbers are listed in Supplementary material 1. The data that supports the findings of this study are available in the supplementary material of this article (Supplementary material 1 and Supplementary material 2).
