# Supplementary material for: Soil disturbance influences ectomycorrhizal fungal communities: an in-situ experiment under Mediterranean holm oak (Quercus ilex L.)
Source: Mycorrhiza. 2026 Aug 13;36(4):58. doi: 10.1007/s00572-026-01302-2 (PMC13469222; doi:10.1007/s00572-026-01302-2)
Supplement: Supplementary file 1 [file 572_2026_1302_MOESM1_ESM.docx]

**Supplementary material 1** Abundance (number of root tips) at Vic (V), Puechabon (P) and Les Mages (LM) in 2009 and 2010 by exploration strategy (contact (C), short-distance (S), medium-distance (M) and long-distance (L))

| **Year** | **Site** | **Depth** | **Core** | **C** | **S** | **M** | **L** | **Abundance** |
| --- | --- | --- | --- | --- | --- | --- | --- | --- |
| 2009 | V | a | 1 | 12 | 0 | 1 | 0 | 13 |
| 2009 | V | a | 2 | 11 | 0 | 6 | 0 | 17 |
| 2009 | V | a | 3 | 5 | 0 | 0 | 0 | 5 |
| 2009 | V | a | 6 | 4 | 0 | 0 | 0 | 4 |
| 2009 | V | a | 8 | 20 | 0 | 0 | 0 | 20 |
| 2009 | V | a | 9 | 5 | 0 | 0 | 0 | 5 |
| 2009 | V | a | 11 | 5 | 1 | 1 | 0 | 7 |
| 2009 | V | a | 12 | 12 | 3 | 0 | 0 | 15 |
| 2009 | V | a | 13 | 20 | 0 | 0 | 0 | 20 |
| 2009 | V | a | 14 | 2 | 0 | 0 | 0 | 2 |
| 2009 | V | a | 15 | 2 | 0 | 0 | 0 | 2 |
| 2009 | V | a | 16 | 2 | 0 | 0 | 0 | 2 |
| 2009 | V | a | 17 | 12 | 0 | 0 | 0 | 12 |
| 2009 | V | a | 18 | 5 | 0 | 0 | 0 | 5 |
| 2009 | V | a | 20 | 1 | 0 | 1 | 0 | 2 |
| 2009 | V | a | 22 | 20 | 0 | 0 | 0 | 20 |
| 2009 | V | a | 23 | 18 | 0 | 0 | 0 | 18 |
| 2009 | V | a | 25 | 6 | 0 | 1 | 0 | 7 |
| 2009 | V | b | 2 | 4 | 4 | 2 | 0 | 10 |
| 2009 | V | b | 4 | 9 | 0 | 0 | 0 | 9 |
| 2009 | V | b | 7 | 1 | 0 | 0 | 0 | 1 |
| 2009 | V | b | 8 | 2 | 0 | 0 | 0 | 2 |
| 2009 | V | b | 11 | 7 | 0 | 1 | 0 | 8 |
| 2009 | V | b | 13 | 4 | 1 | 0 | 0 | 5 |
| 2009 | V | b | 15 | 1 | 0 | 0 | 0 | 1 |
| 2009 | V | b | 16 | 2 | 0 | 0 | 0 | 2 |
| 2009 | V | b | 18 | 4 | 0 | 0 | 0 | 4 |
| 2009 | V | b | 22 | 2 | 0 | 0 | 0 | 2 |
| 2009 | V | b | 23 | 8 | 0 | 0 | 0 | 8 |
| 2009 | P | a | 1 | 20 | 0 | 0 | 0 | 20 |
| 2009 | P | a | 1 | 12 | 0 | 0 | 0 | 12 |
| 2009 | P | a | 2 | 18 | 1 | 1 | 0 | 20 |
| 2009 | P | a | 2 | 18 | 2 | 0 | 0 | 20 |
| 2009 | P | a | 3 | 20 | 0 | 0 | 0 | 20 |
| 2009 | P | a | 3 | 19 | 1 | 0 | 0 | 20 |
| 2009 | P | a | 4 | 20 | 0 | 0 | 0 | 20 |
| 2009 | P | a | 4 | 20 | 0 | 0 | 0 | 20 |
| 2009 | P | a | 5 | 18 | 0 | 2 | 0 | 20 |
| 2009 | P | a | 5 | 15 | 3 | 2 | 0 | 20 |
| 2009 | P | a | 6 | 20 | 0 | 0 | 0 | 20 |
| 2009 | P | a | 6 | 17 | 0 | 3 | 0 | 20 |
| 2009 | P | a | 7 | 20 | 0 | 0 | 0 | 20 |
| 2009 | P | a | 7 | 20 | 0 | 0 | 0 | 20 |
| 2009 | P | a | 8 | 20 | 0 | 0 | 0 | 20 |
| 2009 | P | a | 9 | 10 | 0 | 0 | 0 | 10 |
| 2009 | P | a | 1 | 3 | 2 | 1 | 0 | 6 |
| 2009 | P | a | 2 | 1 | 4 | 0 | 0 | 5 |
| 2009 | P | a | 3 | 11 | 3 | 1 | 2 | 17 |
| 2009 | P | b | 1 | 9 | 0 | 1 | 0 | 10 |
| 2009 | P | b | 2 | 14 | 1 | 0 | 0 | 15 |
| 2009 | P | b | 3 | 19 | 0 | 0 | 0 | 19 |
| 2009 | P | b | 4 | 13 | 1 | 0 | 0 | 14 |
| 2009 | P | b | 5 | 1 | 0 | 1 | 0 | 2 |
| 2009 | P | b | 7 | 3 | 0 | 0 | 0 | 3 |
| 2009 | P | b | 7 | 3 | 0 | 0 | 0 | 3 |
| 2009 | P | b | 9 | 7 | 0 | 0 | 0 | 7 |
| 2009 | LM | a | 1 | 15 | 4 | 1 | 0 | 20 |
| 2009 | LM | a | 2 | 16 | 2 | 2 | 0 | 20 |
| 2009 | LM | a | 3 | 20 | 0 | 0 | 0 | 20 |
| 2009 | LM | a | 4 | 20 | 0 | 0 | 0 | 20 |
| 2009 | LM | a | 5 | 20 | 0 | 0 | 0 | 20 |
| 2009 | LM | a | 6 | 12 | 6 | 2 | 0 | 20 |
| 2009 | LM | a | 7 | 20 | 0 | 0 | 0 | 20 |
| 2009 | LM | a | 8 | 12 | 1 | 4 | 3 | 20 |
| 2009 | LM | a | 9 | 10 | 9 | 1 | 0 | 20 |
| 2009 | LM | a | 10 | 19 | 0 | 1 | 0 | 20 |
| 2009 | LM | a | 11 | 18 | 0 | 0 | 2 | 20 |
| 2009 | LM | a | 12 | 18 | 0 | 0 | 2 | 20 |
| 2009 | LM | a | 13 | 6 | 7 | 5 | 0 | 18 |
| 2009 | LM | a | 14 | 20 | 0 | 0 | 0 | 20 |
| 2009 | LM | a | 15 | 20 | 0 | 0 | 0 | 20 |
| 2009 | LM | a | 16 | 17 | 3 | 0 | 0 | 20 |
| 2009 | LM | a | 17 | 17 | 3 | 0 | 0 | 20 |
| 2009 | LM | a | 18 | 20 | 0 | 0 | 0 | 20 |
| 2009 | LM | a | 19 | 20 | 0 | 0 | 0 | 20 |
| 2009 | LM | a | 20 | 19 | 1 | 0 | 0 | 20 |
| 2009 | LM | a | 21 | 19 | 0 | 1 | 0 | 20 |
| 2009 | LM | a | 22 | 17 | 1 | 2 | 0 | 20 |
| 2009 | LM | a | 23 | 16 | 2 | 2 | 0 | 20 |
| 2009 | LM | a | 24 | 18 | 2 | 0 | 0 | 20 |
| 2009 | LM | a | 25 | 20 | 0 | 0 | 0 | 20 |
| 2009 | LM | b | 2 | 8 | 0 | 2 | 0 | 10 |
| 2009 | LM | b | 4 | 19 | 0 | 1 | 0 | 20 |
| 2009 | LM | b | 6 | 1 | 0 | 2 | 0 | 3 |
| 2009 | LM | b | 8 | 2 | 0 | 0 | 0 | 2 |
| 2009 | LM | b | 9 | 9 | 5 | 4 | 2 | 20 |
| 2009 | LM | b | 12 | 16 | 1 | 0 | 0 | 17 |
| 2009 | LM | b | 13 | 0 | 0 | 0 | 20 | 20 |
| 2009 | LM | b | 16 | 7 | 3 | 0 | 0 | 10 |
| 2009 | LM | b | 17 | 8 | 8 | 4 | 0 | 20 |
| 2009 | LM | b | 20 | 6 | 11 | 3 | 0 | 20 |
| 2009 | LM | b | 21 | 10 | 0 | 5 | 0 | 15 |
| 2009 | LM | b | 23 | 5 | 0 | 1 | 0 | 6 |
| 2009 | LM | b | 25 | 4 | 0 | 16 | 0 | 20 |
| 2010 | 600 | a | 1 | 20 | 0 | 0 | 0 | 20 |
| 2010 | 600 | a | 3 | 16 | 1 | 2 | 0 | 19 |
| 2010 | 600 | a | 4 | 5 | 15 | 0 | 0 | 20 |
| 2010 | 600 | a | 5 | 7 | 1 | 0 | 0 | 8 |
| 2010 | V | a | 6 | 7 | 6 | 3 | 0 | 16 |
| 2010 | V | a | 7 | 14 | 1 | 1 | 0 | 16 |
| 2010 | V | a | 8 | 17 | 2 | 0 | 0 | 19 |
| 2010 | V | a | 9 | 21 | 0 | 0 | 0 | 20 |
| 2010 | V | a | 10 | 12 | 8 | 0 | 0 | 20 |
| 2010 | V | a | 11 | 18 | 2 | 0 | 0 | 20 |
| 2010 | V | a | 12 | 18 | 2 | 0 | 0 | 20 |
| 2010 | V | a | 13 | 19 | 0 | 1 | 0 | 20 |
| 2010 | V | a | 14 | 7 | 0 | 0 | 0 | 7 |
| 2010 | V | a | 15 | 12 | 0 | 0 | 0 | 12 |
| 2010 | V | a | 16 | 12 | 0 | 0 | 0 | 12 |
| 2010 | V | a | 17 | 9 | 2 | 1 | 0 | 12 |
| 2010 | V | a | 18 | 6 | 0 | 0 | 0 | 6 |
| 2010 | V | a | 19 | 8 | 1 | 0 | 0 | 9 |
| 2010 | V | a | 20 | 6 | 2 | 1 | 0 | 9 |
| 2010 | V | a | 21 | 22 | 0 | 0 | 0 | 20 |
| 2010 | V | a | 22 | 14 | 6 | 0 | 0 | 20 |
| 2010 | V | a | 23 | 10 | 2 | 0 | 0 | 12 |
| 2010 | V | a | 24 | 15 | 0 | 1 | 0 | 16 |
| 2010 | V | a | 25 | 5 | 0 | 0 | 0 | 5 |
| 2010 | V | b | 4 | 8 | 0 | 0 | 0 | 8 |
| 2010 | V | b | 7 | 19 | 1 | 0 | 0 | 20 |
| 2010 | V | b | 8 | 5 | 1 | 0 | 0 | 6 |
| 2010 | V | b | 9 | 17 | 3 | 0 | 0 | 20 |
| 2010 | V | b | 11 | 20 | 0 | 0 | 0 | 20 |
| 2010 | V | b | 13 | 18 | 2 | 0 | 0 | 20 |
| 2010 | V | b | 15 | 8 | 1 | 1 | 0 | 10 |
| 2010 | V | b | 16 | 5 | 0 | 0 | 0 | 5 |
| 2010 | V | b | 18 | 8 | 4 | 0 | 0 | 12 |
| 2010 | V | b | 22 | 8 | 7 | 1 | 0 | 16 |
| 2010 | V | b | 23 | 23 | 1 | 0 | 0 | 20 |
| 2010 | V | b | 25 | 20 | 0 | 0 | 0 | 20 |
| 2010 | P | a | 1 | 18 | 1 | 1 | 0 | 20 |
| 2010 | P | a | 2 | 11 | 4 | 5 | 0 | 20 |
| 2010 | P | a | 3 | 13 | 7 | 0 | 0 | 20 |
| 2010 | P | a | 4 | 16 | 4 | 0 | 0 | 20 |
| 2010 | P | a | 6 | 6 | 8 | 2 | 0 | 16 |
| 2010 | P | a | 7 | 15 | 5 | 0 | 0 | 20 |
| 2010 | P | a | 8 | 14 | 4 | 2 | 0 | 20 |
| 2010 | P | a | 9 | 9 | 7 | 4 | 0 | 20 |
| 2010 | P | b | 1 | 12 | 5 | 3 | 0 | 20 |
| 2010 | P | b | 3 | 8 | 2 | 10 | 0 | 20 |
| 2010 | P | b | 5 | 19 | 0 | 1 | 0 | 20 |
| 2010 | P | b | 7 | 12 | 5 | 3 | 0 | 20 |
| 2010 | P | b | 9 | 8 | 11 | 2 | 0 | 20 |
| 2010 | P | a | 1 | 14 | 6 | 0 | 0 | 20 |
| 2010 | P | a | 2 | 8 | 10 | 2 | 0 | 20 |
| 2010 | P | a | 3 | 8 | 8 | 3 | 1 | 20 |
| 2010 | P | a | 5 | 13 | 2 | 4 | 0 | 19 |
| 2010 | P | a | 6 | 0 | 17 | 3 | 0 | 20 |
| 2010 | P | a | 7 | 9 | 6 | 5 | 0 | 20 |
| 2010 | P | b | 2 | 18 | 0 | 2 | 0 | 20 |
| 2010 | P | b | 7 | 14 | 5 | 1 | 0 | 20 |
| 2010 | LM | a | 1 | 3 | 16 | 1 | 0 | 20 |
| 2010 | LM | a | 2 | 12 | 7 | 1 | 0 | 20 |
| 2010 | LM | a | 3 | 0 | 19 | 1 | 0 | 20 |
| 2010 | LM | a | 4 | 15 | 4 | 1 | 0 | 20 |
| 2010 | LM | a | 5 | 11 | 2 | 3 | 0 | 16 |
| 2010 | LM | a | 6 | 13 | 6 | 1 | 0 | 20 |
| 2010 | LM | a | 7 | 9 | 3 | 8 | 0 | 20 |
| 2010 | LM | a | 8 | 8 | 1 | 11 | 0 | 20 |
| 2010 | LM | a | 9 | 14 | 1 | 1 | 0 | 16 |
| 2010 | LM | a | 10 | 7 | 2 | 4 | 0 | 13 |
| 2010 | LM | a | 11 | 17 | 1 | 2 | 0 | 20 |
| 2010 | LM | a | 12 | 17 | 2 | 1 | 0 | 20 |
| 2010 | LM | a | 15 | 14 | 2 | 4 | 0 | 20 |
| 2010 | LM | a | 16 | 13 | 4 | 3 | 0 | 20 |
| 2010 | LM | a | 17 | 8 | 5 | 3 | 0 | 16 |
| 2010 | LM | a | 18 | 18 | 0 | 2 | 0 | 20 |
| 2010 | LM | a | 19 | 18 | 0 | 2 | 0 | 20 |
| 2010 | LM | a | 20 | 13 | 5 | 1 | 1 | 20 |
| 2010 | LM | a | 21 | 14 | 5 | 1 | 0 | 20 |
| 2010 | LM | a | 22 | 14 | 3 | 3 | 0 | 20 |
| 2010 | LM | a | 23 | 9 | 5 | 3 | 3 | 20 |
| 2010 | LM | a | 24 | 9 | 3 | 8 | 0 | 20 |
| 2010 | LM | a | 25 | 6 | 2 | 12 | 0 | 20 |
| 2010 | LM | b | 2 | 12 | 4 | 4 | 0 | 20 |
| 2010 | LM | b | 4 | 2 | 14 | 4 | 0 | 20 |
| 2010 | LM | b | 6 | 9 | 2 | 0 | 0 | 11 |
| 2010 | LM | b | 8 | 16 | 0 | 8 | 0 | 20 |
| 2010 | LM | b | 9 | 12 | 7 | 5 | 0 | 20 |
| 2010 | LM | b | 12 | 14 | 4 | 2 | 0 | 20 |
| 2010 | LM | b | 16 | 12 | 3 | 1 | 0 | 16 |
| 2010 | LM | b | 17 | 14 | 1 | 2 | 0 | 17 |
| 2010 | LM | b | 20 | 12 | 3 | 5 | 0 | 20 |
| 2010 | LM | b | 21 | 11 | 2 | 2 | 0 | 15 |
| 2010 | LM | b | 23 | 3 | 4 | 13 | 0 | 20 |
| 2010 | LM | b | 25 | 10 | 0 | 1 | 0 | 11 |

**Supplementary material 2** Abundance (number of root tips) at Vic (V), Puechabon (P) and Les Mages (LM) in 2009 and 2010 for the 103 OTUs, with closest GenBank matches, best UNITE Species Hypothesis, and accession numbers

| **Taxon** | **V2009** | **V2010** | **P2009** | **P2010** | **LM2009** | **LM2010** | **Closest Genbank sequence (% identity) ^1^** | **Best UNITE Species Hypothesis (% threshold) ^2^** | **GenBank Id ^3^** |
| --- | --- | --- | --- | --- | --- | --- | --- | --- | --- |
| *Cadophora* sp. | 0 | 0 | 1 | 0 | 0 | 0 | KF887110 (91.0%) | Sequence absent from UNITE | JF506855* |
| *Cenococcum geophilum* | 2 | 2 | 2 | 6 | 12 | 8 | MK285742 (99.1%) | SH0999694.10FU (1.5%) | JF506744 |
| *Ceratobasidium* sp. | 2 | 0 | 0 | 0 | 0 | 0 | JF506743 (99.9%) | SH0155272.10FU (3%) | JF506742 |
| *Clavulina* sp. | 0 | 0 | 2 | 0 | 4 | 0 | PV135132 (97.9%) | SH0082671.10FU (3%) | JF506745 |
| *Cortinarius epipurrus* | 0 | 0 | 0 | 0 | 3 | 0 | PQ639012 (99.7%) | SH0674964.10FU (2%) | JF506767 |
| *Cortinarius intempestivus* | 0 | 0 | 0 | 0 | 1 | 0 | PX369447 (99.8%) | SH1365078.10FU (1%) | JF506769 |
| *Cortinarius pseudocollinitus* | 2 | 0 | 0 | 0 | 0 | 0 | GU256203 (99.8%) | SH1017863.10FU (1.5%) | JF506747 |
| *Cortinarius puellaris* | 0 | 0 | 0 | 0 | 1 | 0 | NR_153052 (98.1%) | SH0986103.10FU (1.5%) | JF506768 |
| *Cortinarius* cf. *pulchripes* | 0 | 0 | 1 | 0 | 0 | 0 | OP684917 (93.2%) | Sequence absent from UNITE | JF506752* |
| *Cortinarius* sp.1 (Obtusi) | 0 | 0 | 2 | 0 | 0 | 0 | KM576362 (98.7%) | SH0701549.10FU (2%) | JF506749 |
| *Cortinarius* sp.2 (Obtusi) | 3 | 0 | 0 | 0 | 0 | 0 | KP889728 (94.3%) | SH0192047.10FU (3%) | JF506750 |
| *Cortinarius* sp.3 (Obtusi) | 0 | 0 | 0 | 0 | 1 | 0 | KU973875 (96.3%) | Sequence absent from UNITE | JF506748* |
| *Cortinarius* cf. *subturibulosus* | 0 | 0 | 0 | 0 | 1 | 0 | JF506752 (87.4%) | SH0198430.10FU (3%) | JF506751* |
| *Craterellus sinuosus* | 0 | 0 | 0 | 0 | 8 | 0 | FR852287 (97.7%) | SH0351139.10FU (2.5%) | JF506753* |
| *Craterellus* sp. | 0 | 0 | 0 | 0 | 1 | 0 | KC791143 (96.6%) | SH0124845.10FU (3%) | PX763668* |
| *Elaphomyces muricatus* | 0 | 0 | 0 | 0 | 1 | 0 | OQ750218 (99.5%) | SH0167678.10FU (3%) | JF506754 |
| *Entoloma platyphylloides* | 0 | 0 | 0 | 0 | 1 | 0 | MZ868960 (99.8%) | SH0311436.10FU (2.5%) | JF506755 |
| *Genea pinicola* | 0 | 0 | 1 | 0 | 0 | 0 | NR_155120 (99.4%) | SH0105311.10FU (3%) | PX763669 |
| *Genea* sp. | 0 | 0 | 1 | 1 | 0 | 0 | NA | SH0200337.10FU (3%) | JF506842* |
| *Hebeloma* cf. *quercetorum* | 0 | 0 | 0 | 0 | 1 | 0 | FR852300 (82.4%) | Sequence absent from UNITE | JF506758* |
| *Hebeloma sinapizans* | 0 | 0 | 0 | 0 | 1 | 5 | OL461970 (100%) | SH1903330.10FU (0.5%) | JF506756 |
| *Helvella acetabulum* | 0 | 3 | 0 | 0 | 0 | 0 | OR159643 (99.1%) | SH0107092.10FU (3%) | PX763670 |
| *Helvella crispa* aff. | 0 | 0 | 0 | 0 | 0 | 5 | KU739871 (96.0%) | NA | JF506760* |
| *Helvella neopallescens* | 0 | 3 | 0 | 8 | 2 | 11 | KR819045 (99.5%) | SH0102307.10FU (3%) | JF506761 |
| *Helvellosebacina* sp.1 | 0 | 0 | 0 | 0 | 2 | 0 | PV719311 (95.0%) | SH0107205.10FU (3%) | JF506803 |
| *Helvellosebacina* sp.2 | 0 | 0 | 0 | 0 | 1 | 5 | FJ946950 (96.9%) | SH0107203.10FU (3%) | JF506804 |
| *Humaria* sp.1 | 1 | 5 | 0 | 0 | 2 | 0 | AY351626 (99.2%) | SH0745730.10FU (1.5%) | JF506762 |
| *Humaria* sp.2 | 0 | 2 | 0 | 0 | 2 | 5 | OR159622 (99.3%) | SH0011311.10FU (3%) | PX763690 |
| *Hygrophorus roseodiscoideus* | 1 | 0 | 0 | 0 | 0 | 0 | MZ576442 (99.6%) | SH0170041.10FU (3%) | JF506763 |
| *Hygrophorus russula* | 6 | 0 | 7 | 0 | 0 | 0 | MW290205 (100%) | SH0193842.10FU (3%) | JF506764 |
| *Hymenogaster* sp. | 0 | 0 | 1 | 0 | 0 | 0 | MG274235 (96.1%) | Sequence absent from UNITE | JF506765* |
| I*nocybe griseolilacina* | 1 | 0 | 4 | 0 | 2 | 1 | JN580886 (98.8%) | SH0156063.10FU (3%) | JF506766 |
| *Inocybe mycenoides* | 0 | 0 | 4 | 0 | 0 | 0 | FR852249 (99.8%) | SH0955444.10FU (1.5%) | JF506770 |
| *Inocybe tenebrosa* | 1 | 0 | 1 | 0 | 0 | 0 | MW027959 (100%) | SH0902050.10FU (1.5%) | JF506772 |
| *Inocybe* aff. *tigrina* | 0 | 0 | 1 | 1 | 0 | 0 | KP826720 (100%) | SH0685367.10FU (2%) | JF506771 |
| *Lactarius acerrimus* | 0 | 0 | 4 | 0 | 0 | 0 | KX610689 (99.5%) | Sequence absent from UNITE | JF506775 |
| *Lactarius decipiens* | 2 | 0 | 2 | 0 | 17 | 0 | KM576494 (99.3%) | SH1840532.10FU (0.5%) | PX763671 |
| *Lactarius mediterraneensis* | 1 | 0 | 1 | 0 | 0 | 0 | JF908267 (99.9%) | SH1333103.10FU (1%) | JF506777 |
| *Lactarius* cf. *zonarius* | 0 | 0 | 0 | 0 | 5 | 0 | MW554121 (93.9%) | SH0210176.10FU (3%) | JF506776* |
| *Lactifluus* cf. *vellereus* | 0 | 0 | 0 | 0 | 43 | 0 | MN265590 (96.7%) | SH0171873.10FU (3%) | JF506778* |
| *Leccinellum lepidum* | 0 | 0 | 0 | 0 | 1 | 0 | MH011879 (99.6%) | SH0002219.10FU (3%) | JF506779 |
| *Membranomyces* sp. | 1 | 0 | 1 | 0 | 0 | 0 | GQ254855 (98.7%) | SH0224755.10FU (2.5%) | PX763672 |
| *Paragalactinia* cf. *hypoleuca* | 0 | 3 | 0 | 0 | 0 | 0 | OQ418569 (90.5%) | Sequence absent from UNITE | JF506781* |
| *Paragalactinia michelii* | 0 | 0 | 0 | 0 | 0 | 2 | JX989985 (99%) | SH0952904.10FU (1.5%) | JF506780 |
| *Peziza saccardoana* | 0 | 0 | 0 | 0 | 0 | 2 | PP278012 (99.2%) | SH0284757.10FU (2.5%) | PX763689 |
| *Polyphilus* sp. | 0 | 0 | 0 | 0 | 1 | 0 | JN859270 (99.1%) | SH1268646.10FU (1%) | JF506850 |
| *Russula* cf. *alutacea* | 15 | 0 | 6 | 0 | 0 | 0 | MG680185 (99.5%) | SH0938729.10FU (1.5%) | PX763675 |
| *Russula decipiens* | 0 | 0 | 4 | 0 | 12 | 0 | OR159529 (100%) | SH0956499.10FU (1.5%) | PX763676 |
| *Russula* aff. *delica* | 0 | 0 | 0 | 0 | 4 | 0 | HF565098 (100%) | SH0586119.10FU (2%) | PX763673 |
| *Russula foetens* | 0 | 0 | 0 | 0 | 9 | 0 | PQ652660 (100%) | SH1158895.10FU (1%) | PX763678 |
| *Russula fragilis* | 0 | 0 | 0 | 0 | 2 | 0 | KM576547 (98.1%) | SH0956398.10FU (1.5%) | PX763679 |
| *Russula galileensis* | 0 | 0 | 2 | 0 | 0 | 0 | EU403089 (99.8%) | SH0650065.10FU (2%) | PX763680 |
| *Russula globispora* | 12 | 0 | 0 | 0 | 3 | 0 | MG687348 (99.6%) | SH0956860.10FU (1.5%) | PX763681 |
| *Russula nympharum* | 6 | 0 | 21 | 0 | 2 | 0 | KU928158 (99.9%) | SH0152549.10FU (3%) | PX763682 |
| *Russula occasus* | 2 | 0 | 0 | 0 | 0 | 0 | PV834989 (99.7%) | SH0650441.10FU (2%) | PX763674 |
| *Russula* aff. *pelargonia* | 0 | 0 | 0 | 0 | 1 | 0 | PV742600 (92.9%) | SH0152063.10FU (3%) | JF506787* |
| *Russula putida* | 0 | 0 | 0 | 0 | 5 | 0 | JF908679 (99.2%) | SH0817350.10FU (1.5%) | PX763677 |
| *Russula quercilicis* | 15 | 0 | 0 | 0 | 0 | 0 | MT011386 (99.4%) | SH0152079.10FU (3%) | PX763684 |
| *Russula* aff. *vinosobrunnea* | 1 | 0 | 0 | 0 | 22 | 0 | MW028086 (98.4%) | SH0635155.10FU (2%) | PX763683 |
| *Sarcosphaera crassa* | 0 | 0 | 0 | 0 | 0 | 2 | PV690308 (99.8%) | SH0639138.10FU (2%) | PX763685 |
| *Sebacina* cf. *incrustans* | 2 | 30 | 1 | 1 | 0 | 1 | EF644113 (98.1%) | SH0934125.10FU (1.5%) | JF506813 |
| *Sebacina* sp.1 | 0 | 0 | 1 | 4 | 0 | 0 | MN970756 (96.7%) | SH0630925.10FU (2%) | JF506811 |
| *Sebacina* sp.2 | 0 | 6 | 0 | 0 | 2 | 0 | AY452678 (98.0%) | SH0369583.10FU (2.5%) | JF506805 |
| *Sebacina* sp.3 | 0 | 0 | 0 | 0 | 1 | 0 | FN669251 (96.6%) | SH0631035.10FU (2%) | JF506806 |
| *Sebacina* sp.4 | 0 | 0 | 0 | 0 | 1 | 8 | LC547702 (97.0%) | SH0136871.10FU (3%) | JF506810 |
| *Sebacina* sp.5 | 0 | 0 | 1 | 0 | 0 | 0 | EF218815 (96.4%) | SH0136782.10FU (3%) | JF506808 |
| *Sebacina* sp.6 | 0 | 0 | 0 | 0 | 2 | 0 | FR852337 (95.2%) | SH0136589.10FU (3%) | JF506809 |
| *Sebacina* sp.7 | 0 | 0 | 0 | 0 | 0 | 7 | KF000575 (97.5%) | SH0631189.10FU (2%) | JF506812 |
| *Sebacina* sp.8 | 0 | 0 | 0 | 0 | 0 | 1 | MN818547 (94.1%) | NA | JF506807* |
| *Sebacina* sp.9 | 0 | 12 | 0 | 0 | 0 | 1 | NA | Sequence absent from UNITE | JF506853* |
| *Sistotrema pistilliferum* | 2 | 0 | 6 | 0 | 2 | 0 | HQ204634 (98.7%) | SH0184487.10FU (3%) | JF506746 |
| *Tarzetta cupularis* | 0 | 0 | 2 | 0 | 0 | 2 | HQ204696 (99.2%) | SH0132063.10FU (3%) | JF506847 |
| *Tarzetta ochracea* | 0 | 0 | 0 | 2 | 0 | 0 | JX625301 (99.6%) | SH0167206.10FU (3%) | JF506848 |
| *Tarzetta quercus-ilicis* | 0 | 0 | 0 | 0 | 0 | 1 | HE687130 (99.2%) | SH0005017.10FU (3%) | JF506849 |
| *Thelephora pallidocastanea* | 0 | 0 | 0 | 0 | 1 | 0 | LC712245 (99.7%) | SH0618351.10FU (2%) | PX763687 |
| *Thelephora* sp.1 | 4 | 0 | 0 | 2 | 1 | 0 | MF352800 (99.5%) | SH1764915.10FU (0.5%) | JF506827 |
| *Thelephora* sp.2 | 0 | 2 | 1 | 0 | 0 | 0 | MW027975 (99.4%) | SH1280460.10FU (1%) | JF506830 |
| *Thelephora* sp.3 | 1 | 0 | 1 | 0 | 0 | 0 | KM247738 (99.7%) | SH0918653.10FU (1.5%) | JF506816 |
| *Thelephora* sp.4 | 1 | 0 | 1 | 0 | 0 | 0 | KM247766 (97.6%) | SH0128520.10FU (3%) | JF506821 |
| *Thelephora* sp.5 | 1 | 0 | 0 | 0 | 0 | 0 | JX625343 (99.0%) | SH0125265.10FU (3%) | JF506831 |
| *Thelephora* sp.6 | 0 | 0 | 0 | 0 | 3 | 0 | OR159575 (100%) | SH0618650.10FU (2%) | JF506822 |
| *Thelephora* sp.7 | 0 | 4 | 0 | 1 | 0 | 0 | HQ204725 (99.1%) | Sequence absent from UNITE | JF506815* |
| *Thelephora* sp.8 | 0 | 0 | 0 | 0 | 0 | 4 | AY940648 (99.8%) | SH0619601.10FU (2%) | JF506832 |
| *Thelephora* sp.9 | 0 | 0 | 1 | 0 | 0 | 0 | FJ210760 (97.6%) | SH1766688.10FU (0.5%) | PX763686 |
| *Thelephora* sp.10 | 0 | 0 | 0 | 0 | 1 | 3 | KX510034 (98.0%) | SH0126819.10FU (3%) | JF506825 |
| *Thelephora* sp.11 | 0 | 0 | 0 | 0 | 1 | 0 | GU220639 (96.7%) | SH0126821.10FU (3%) | JF506828 |
| *Thelephora* sp.12 | 0 | 0 | 1 | 0 | 0 | 0 | KM247721 (98.1%) | SH0128442.10FU (3%) | JF506820 |
| *Thelephora* sp.13 | 3 | 0 | 0 | 0 | 0 | 0 | EU668943 (99.1%) | SH0126524.10FU (3%) | JF506823 |
| *Thelephora* sp.14 | 0 | 0 | 0 | 0 | 1 | 0 | FJ897240 (98.9%) | SH0128614.10FU (3%) | JF506818 |
| *Thelephora* sp.15 | 0 | 0 | 0 | 0 | 1 | 0 | MK285889 (100%) | SH1282072.10FU (1%) | JF506817 |
| *Thelephora* sp.16 | 0 | 1 | 0 | 0 | 0 | 0 | JX316246 (99.7%) | SH1280537.10FU (1%) | JF506829 |
| *Thelephora* sp.17 | 2 | 0 | 0 | 0 | 1 | 0 | AJ893310 (98.6%) | SH0617096.10FU (2%) | JF506824 |
| *Thelephora* sp.18 | 0 | 0 | 0 | 7 | 0 | 4 | AJ421251 (100%) | SH0920977.10FU (1.5%) | JF506834 |
| *Thelephora* sp.19 | 0 | 0 | 1 | 0 | 0 | 0 | MW027965 (99.5%) | SH0128514.10FU (3%) | JF506814 |
| *Thelephora* sp.20 | 0 | 0 | 1 | 0 | 0 | 0 | KM576631 (100%) | SH0126648.10FU (3%) | JF506819 |
| *Thelephora* sp.21 | 0 | 0 | 2 | 0 | 0 | 0 | LC623427 (97.5%) | SH0127857.10FU (3%) | JF506826 |
| *Tricholoma* cf. *argyraceum* | 0 | 0 | 0 | 0 | 1 | 0 | LT000127 (80.5%) | NA | JF506835* |
| *Trichophaea* sp.1 | 2 | 4 | 0 | 0 | 2 | 2 | MW546603 (94.4%) | SH0118619.10FU (3%) | JF506841 |
| *Trichophaea* sp.2 | 0 | 12 | 0 | 0 | 5 | 3 | HQ204750 (100%) | SH0608853.10FU (2%) | JF506836 |
| *Tuber borchii* | 1 | 1 | 0 | 0 | 2 | 1 | KT067681 (100%) | SH0843840.10FU (1.5%) | JF506837 |
| *Tuber* aff. *borchii* | 0 | 0 | 0 | 0 | 2 | 1 | OM002613 (97.7%) | SH0843845.10FU (1.5%) | PX763688 |
| *Tuber dryophilum* | 0 | 1 | 0 | 9 | 0 | 0 | JX844780 (99.0%) | SH0101596.10FU (3%) | JF506839 |
| *Tuber ferrugineum* | 2 | 2 | 0 | 3 | 0 | 0 | FM205601 (99.7%) | SH0148388.10FU (3%) | JF506838 |

^1^ only BLAST hits with query cover > 70% are listed, otherwise NA (not assigned) is indicated.

^2^ NA (not assigned) is indicated for sequences present in UNITE but not associated to any Species Hypotheses.

^3^ sequences with asterisks display quality issues (see Materials & Methods).

**Supplementary material 3** Detailed information on the updated taxonomic annotation of fungal ITS sequences.

In the course of this annotation update, we came across several sequences that failed to match any Genbank sequence with a query cover above 70% or that displayed a max % identity with their best BLAST hit abnormally low (below 95%), or that were absent from UNITE or that formed unique SHs at the 3% distance threshold. Inspection of these sequences revealed obvious quality issues hallmarked by e.g. high frequency of ambiguity letters, concentration of SNPs at sequence ends or presence of SNPs in the 5.8S, lineage-dependent deletion of identical sequence blocks. When these low-quality sequences could be confidently assigned to at least the genus level, they were conserved in the dataset and listed in the last column of Supplementary material 1 with an asterisk. When they confidently fell within an OTU represented by another, better-quality sequence, their occurrence data was considered for the concerned OTU but the sequence was not listed in Supplementary material 1. Eleven previously published sequences had to be removed from our dataset because their uniqueness or conversely, their unambiguous assignment to a represented OTU within the dataset, could not be ascertained (these are JF506784, JF506785, JF506790, JF506791, JF506792, JF506800, JF506802, JF506833, JF506840, JF506851, and JF506854).

**Supplementary material 4** Ratio Asco- and Basidiomycota phyla and relative abundance of EM fungal families in soils collected at the Puéchabon calibration site, according to **Richard et al**. **(2011)**. Numbers in parentheses indicate raw counts

|  | **2007-2008** | | **2008-2009** | |
| --- | --- | --- | --- | --- |
|  | **Autumn** | **Spring** | **Autumn** | **Spring** |
| ‍ |  |  |  |  |
| **Ascomycota/Basidiomycota ratio** | 0.23 | 0.13 | 0.19 | 0.23 |
|  |  |  |  |  |
| Albatrellaceae | 0.29 (1) | 1.75 (6) | 0.38 (1) | 0 (0) |
| Boletaceae | 0 (0) | 0.29 (1) | 0 (0) | 0 (0) |
| Clavulinaceae | 0.58 (2) | 0 (0) | 0.38 (1) | 0 (0) |
| Cortinariaceae | 18.55 (64) | 23.68 (81) | 18.77 (49) | 12.63 (24) |
| Exidiaceae | 3.77 (13) | 3.51 (12) | 9.96 (26) | 7.89 (15) |
| Geastraceae | 0 (0) | 0 (0) | 0.38 (1) | 0.53 (1) |
| Gloniaceae | 12.46 (43) | 8.48 (29) | 14.56 (38) | 12.11 (23) |
| Helvellaceae | 0 (0) | 0.88 (3) | 0.77 (2) | 2.11 (4) |
| Hygrophoraceae | 0.58 (2) | 0.29 (1) | 0.38 (1) | 0 (0) |
| Melanogastraceae | 0 (0) | 0 (0) | 0.38 (1) | 0 (0) |
| Pezizaceae | 3.77 (13) | 0.58 (2) | 0.38 (1) | 0 (0) |
| Pyronemataceae | 0 (0) | 0.58 (2) | 0 (0) | 1.58 (3) |
| Russulaceae | 13.04 (45) | 31.29 (107) | 19.54 (51) | 16.32 (31) |
| Sclerodermataceae | 0.29 (1) | 0 (0) | 1.15 (3) | 0 (0) |
| Thelephoraceae | 43.19 (149) | 27.19 (93) | 27.97 (73) | 45.79 (87) |
| Tricholomataceae | 0.87 (3) | 0.29 (1) | 2.3 (6) | 0.53 (1) |
| Tuberaceae | 1.74 (6) | 0.88 (3) | 0.77 (2) | 0.53 (1) |
| Other Ascomycota | 0.87 (3) | 0.29 (1) | 1.92 (5) | 0 (0) |
| ‍ |  |  |  |  |

**Supplementary material 5** Abundance of ectomycorrhizal (EM) fungal families in soils collected before (black) and after (grey) disturbance across all sites. The number of asterisks refers to the corresponding p-value by chi2 test (*: 0.01 < p-value < 0.05; **: 0.001 < p-value < 0.01; ***: p-value < 0.001)

**Supplementary material 6** Relative abundance of EM fungal families in soils collected before (2009) and after (2010) disturbance across all sites. Numbers in parentheses indicate raw counts

| **Fungal family** | **Relative abundance (%)**  **in 2009 (n)** | **Relative abundance (%)**  **in 2010 (n)** | **Alpha diversity (%)**  **in 2009 (n)** | **Alpha diversity (%)**  **in 2010 (n)** |
| --- | --- | --- | --- | --- |
| Boletaceae | 0.26 (1) | 0 (0) | 1.15 (1) | 0 (0) |
| Ceratobasidiaceae | 0.52 (2) | 0 (0) | 1.15 (1) | 0 (0) |
| Cortinariaceae | 3.87 (15) | 0 (0) | 10.34 (9) | 0 (0) |
| Elaphomycetaceae | 0.26 (1) | 0 (0) | 1.15 (1) | 0 (0) |
| Entolomataceae | 0.26 (1) | 0 (0) | 1.15 (1) | 0 (0) |
| Gloniaceae | 4.12 (16) | 7.31 (16) | 1.15 (1) | 2.63 (1) |
| Helvellaceae | 0.52 (2) | 11.87 (26) | 1.15 (1) | 7.89 (3) |
| Hydnaceae | 6.96 (27) | 0 (0) | 5.75 (5) | 0 (0) |
| Hygrophoraceae | 3.61 (14) | 0 (0) | 2.30 (2) | 0 (0) |
| Hymenogastraceae | 0.77 (3) | 2.28 (5) | 3.45 (3) | 2.63 (1) |
| Inocybaceae | 3.61 (14) | 0.91 (2) | 4.60 (4) | 5.26 (2) |
| Leotiomycetidae | 0.26 (1) | 0 (0) | 1.15 (1) | 0 (0) |
| Pezizaceae | 0 (0) | 4.11 (9) | 0 (0) | 10.53 (4) |
| Ploettnerulaceae | 0.26 (1) | 0 (0) | 1.15 (1) | 0 (0) |
| Pyronemataceae | 4.12 (16) | 15.53 (34) | 6.90 (6) | 13.16 (5) |
| Russulaceae | 56.44 (219) | 0 (0) | 20.69 (18) | 0 (0) |
| Sebacinaceae | 3.61 (14) | 34.70 (76) | 10.34 (9) | 21.05 (8) |
| Tarzettaceae | 0.52 (2) | 2.28 (5) | 1.15 (1) | 7.89 (3) |
| Thelephoraceae | 7.99 (31) | 12.79 (28) | 20.69 (18) | 18.42 (7) |
| Tricholomataceae | 0.26 (1) | 0 (0) | 1,15 (1) | 0 (0) |
| Tuberaceae | 1.80 (7) | 8.22 (18) | 3.45 (3) | 10.53 (4) |

**Supplementary material 7** Abundance of ectomycorrhizal fungal genera in soils collected before (black) and after (grey) soil disturbance across all sites. The number of asterisks refers to the corresponding p-value by chi2 test (*****: 0.05 < p-value < 0.1; *: 0.01 < p-value < 0.05; **: 0.001 < p-value < 0.01; ***: p-value < 0.001)
